# Comprehensive Analysis of Temporal Alterations in Cellular Proteome of *Bacillus subtilis* under Curcumin Treatment

**DOI:** 10.1371/journal.pone.0120620

**Published:** 2015-04-14

**Authors:** Panga Jaipal Reddy, Sneha Sinha, Sandipan Ray, Gajanan J. Sathe, Aditi Chatterjee, T. S. Keshava Prasad, Snigdha Dhali, Rapole Srikanth, Dulal Panda, Sanjeeva Srivastava

**Affiliations:** 1 Department of Biosciences and Bioengineering, Indian Institute of Technology Bombay, Powai, Mumbai, India; 2 Proteomics Laboratory, National Centre for Cell Science, Ganeshkhind, Pune, Maharashtra, India; 3 Institute of Bioinformatics, International Tech Park, Whitefield, Bangalore, India; 4 Manipal University, Madhav Nagar,Manipal, India; George Mason University, UNITED STATES

## Abstract

Curcumin is a natural dietary compound with antimicrobial activity against various gram positive and negative bacteria. This study aims to investigate the proteome level alterations in *Bacillus subtilis* due to curcumin treatment and identification of its molecular/cellular targets to understand the mechanism of action. We have performed a comprehensive proteomic analysis of *B*. *subtilis* AH75 strain at different time intervals of curcumin treatment (20, 60 and 120 min after the drug exposure, three replicates) to compare the protein expression profiles using two complementary quantitative proteomic techniques, 2D-DIGE and iTRAQ. To the best of our knowledge, this is the first comprehensive longitudinal investigation describing the effect of curcumin treatment on *B*. *subtilis* proteome. The proteomics analysis revealed several interesting targets such UDP-N-acetylglucosamine 1-carboxyvinyltransferase 1, putative septation protein SpoVG and ATP-dependent Clp protease proteolytic subunit. Further, *in silico* pathway analysis using DAVID and KOBAS has revealed modulation of pathways related to the fatty acid metabolism and cell wall synthesis, which are crucial for cell viability. Our findings revealed that curcumin treatment lead to inhibition of the cell wall and fatty acid synthesis in addition to differential expression of many crucial proteins involved in modulation of bacterial metabolism. Findings obtained from proteomics analysis were further validated using 5-cyano-2,3-ditolyl tetrazolium chloride (CTC) assay for respiratory activity, resazurin assay for metabolic activity and membrane integrity assay by potassium and inorganic phosphate leakage measurement. The gene expression analysis of selected cell wall biosynthesis enzymes has strengthened the proteomics findings and indicated the major effect of curcumin on cell division.

## Introduction

In spite of worldwide initiatives for the development of a plethora of synthetic and semi-synthetic drugs, emerging drug resistance is still remained as one of the foremost health problems and poses challenges for thriving combat against most of the pathogenic infections [1]. Consequently, there is a growing need for the identification and characterization of new potential drugs from natural and synthetic compounds. Natural products have continued to evolve over thousands of years to counter various pathogenic microbes. Even today, most of the existing antibiotics are derived from the backbone of different natural compounds [2]. *B*. *subtilis* is a widely studied non-pathogenic gram-positive bacterium, which is often used as a model organism for diverse cellular and molecular level studies due to its genetic amenability, availability of complete genome sequence, and easy isolation and culturing procedure.

Curcumin, chemically known as 1,7-bis-(4-hydroxy-3-methoxyphenyl)-1,6-heptadiene-3,5-dione, is a naturally occurring phytochemical obtained from the rhizome of *Curcuma longa*. It is the polyphenolic traditional turmeric powder, which is widely used as a dietary component. Curcumin has anti-tumor [3], anti-oxidant [4], anti-inflammatory [5], anti-genotoxic against the DNA damaging agents [6], phototoxic and photodynamic therapy [7,8], it blocks the cell cycle progression in cancer cells [9] and prevents angiogenesis [10]. Curcumin also possess anti-microbial activity against gram positive and negative bacteria and shows synergetic effects on other drugs in combination therapies [11]. Although, the diverse therapeutic potential of curcumin has been established, its precise mechanism of action and molecular targets in prokaryotic system are mostly obscure. Recently, shikimate pathway, which is essential for aromatic amino acid synthesis has been reported to be a possible targets of curcumin in *Helicobacter pylori* [11]. Interestingly, another study indicates that curcumin can effectively perturb the FtsZ assembly dynamics leading to elongation of the bacterial cell length and reduce the viability [12].

Proteome level analysis is very informative for the identification of molecular targets for development of new antibacterial agents as well as unravelling the mechanism of action of the existing drugs, since most of the drugs act via modification/inhibition of proteins. Proteome analysis of *B*. *subtilis* under various stress conditions, including salt stress [13], glucose starvation [14], thiol-induced stress [15] and different antimicrobial drugs [16] are found to be very enlightening. In the present study, we aimed to decipher the temporal alterations of cellular proteome of *B*. *subtilis* AH75 strain in response to curcumin treatment at three time points (20, 60 and 120 min). Application of two complementary quantitative proteomic techniques; DIGE and iTRAQ in combination with high sensitive mass spectrometry effectively improved the proteome coverage. *In silico* pathway analysis using DAVID and KOBAS revealed modulation of fatty acid biosynthesis, peptidoglycan synthesis/ cell division, respiration and stress response proteins in response to curcumin. In addition, gene expression analysis of cell wall and cell division proteins confirmed the repression of cell wall biosynthesis and division. Multiple functional assays including resazurin microtiter assay for metabolic activity, respiratory activity assay using CTC and measurement of potassium and phosphate release after drug treatment were performed to validate the findings obtained from proteomics analysis. Further, the real-time interaction analysis showed that FtsZ bound to curcumin in concentration dependent manner. This comprehensive proteomic study highlights several interesting targets involved in pathways related to the fatty acid metabolism and cell wall synthesis perturbed by curcumin and contributes to a better understanding of its mode of action, and potential molecular and cellular targets.

## Results

### Effect of Curcumin on *B*. *subtilis* Growth and Cell Morphology

**Fig 1:**
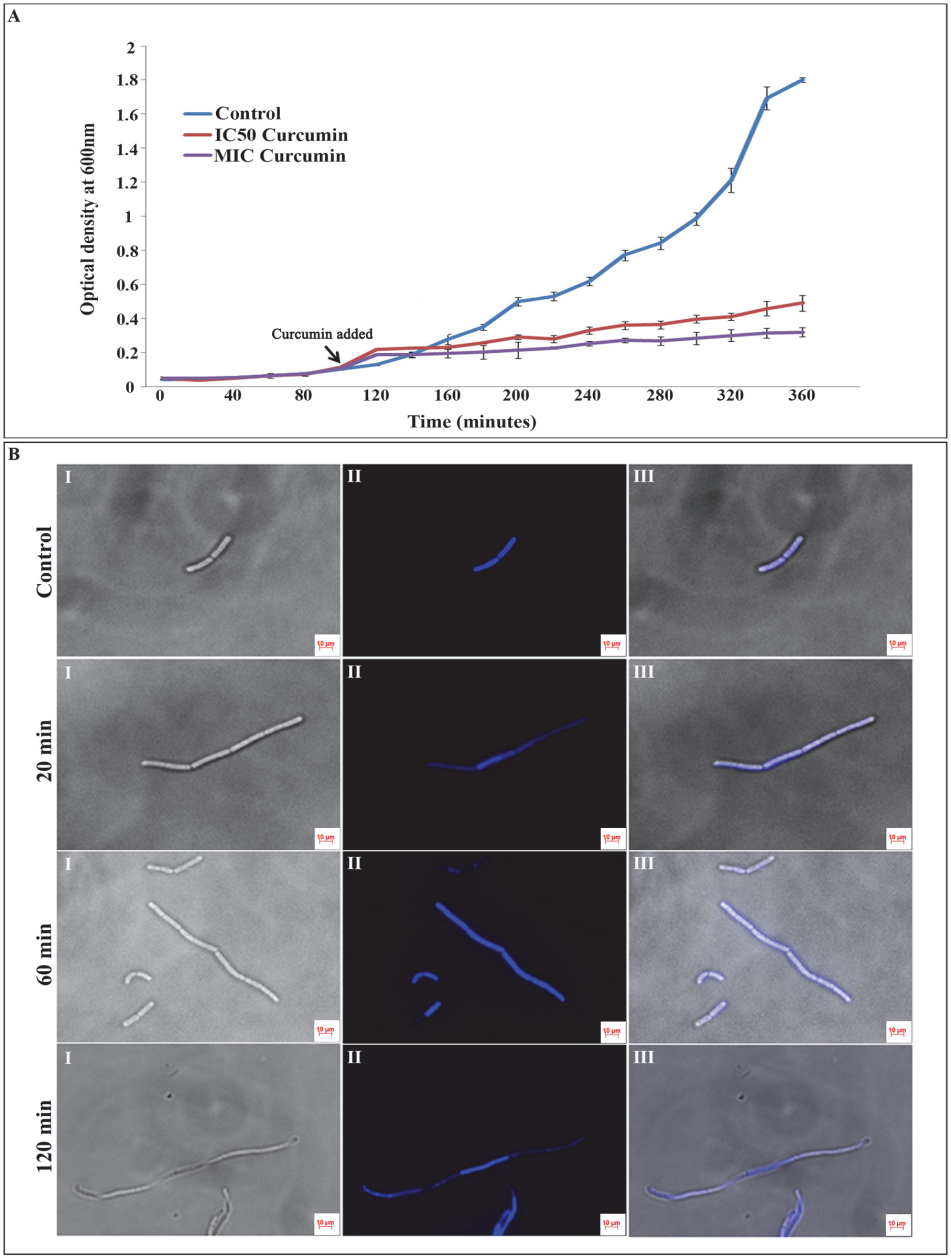
Effect of curcumin treatment on the *B*. *subtilis* AH75 growth and cell morphology. **(A)** *B*. *subtilis* AH75 strain was grown in LB media having spectinomycin antibiotic (100 μg/mL) till the OD_600_ reached to 0.1. Then the cultures were treated with DMSO (control), 20 μM (IC_50_ concentration) and 100 μM (MIC concentration) curcumin. Growth curve was plotted by measuring the OD_600_ for all the samples at every 20 min interval till 360 min (mid-exponential phase). The three time points of curcumin treatment (20, 60 and 120 min) used in proteomic analysis are indicated by arrows. IC_50_ concentration was used for subsequent proteomic analysis. (**B)** *B*. *subtilis* AH75 strain was grown in the presence of 20 μM (IC_50_ concentration) curcumin and the samples was collected after 20, 60 and 120 min of the drug treatment. Cultures treated with only DMSO was used as control. The nuclear materials were stained using 1 μg/μL DAPI for 20 min at room temperature in dark for all the samples. The fluorescence microscopic images were captured with both DAPI and DIC filters. The control *B*. *subtilis* cells showed normal cell length with one or two nucleoids per cell whereas after 20, 60 and 120 min of incubation with 20 μM (IC_50_ concentration) curcumin, most of the cells turned into filamentous structure with multiple nucleoids. I- DIC image, II- DAPI image and III- overlay image.

*B*. *subtilis* growth was measured by calculating the OD_600_ in the presence and absence of the curcumin in three technical replicates (n = 3). The changes in growth pattern for 4 hrs after the addition of the IC_50_ (20 μM) and MIC concentration (100 μM) of the drug have been depicted in the Fig 1A. The growth of the cells treated with 20 μM of curcumin (IC_50_) was significantly declined; whereas cultures treated with 100μM of curcumin (MIC) showed virtually no growth compared to the untreated controls, clearly indicating the antibacterial activity of curcumin against *B*. *subtilis* (Figs 1A and S1A). The comparison of control with and without DMSO indicated no significant change in growth patterns (S1A Fig). Further, the morphological changes in *B*. *subtilis* cells in response to curcumin treatment were investigated using fluorescent microscopy. Untreated *B*. *subtilis* cells (with and without DMSO) showed typical cell morphology with normal size under fluorescent microscopy with single or two nucleoids per cell. Whereas, curcumin (IC_50_-20 μM) treated *B*. *subtilis* cells (20, 60 and 120 min) exhibited significant alterations in cell morphology; filamentous phenotype with multiple nucleoids per cell and a drastic increase in cell length proportionally with the exposure time of the drug (Figs 1B and S1B).

### Effect of Curcumin Treatment on *B*. *subtilis* Proteome Identified in 2D-DIGE and MALDI-TOF/TOF MS Analysis

**Fig 2:**
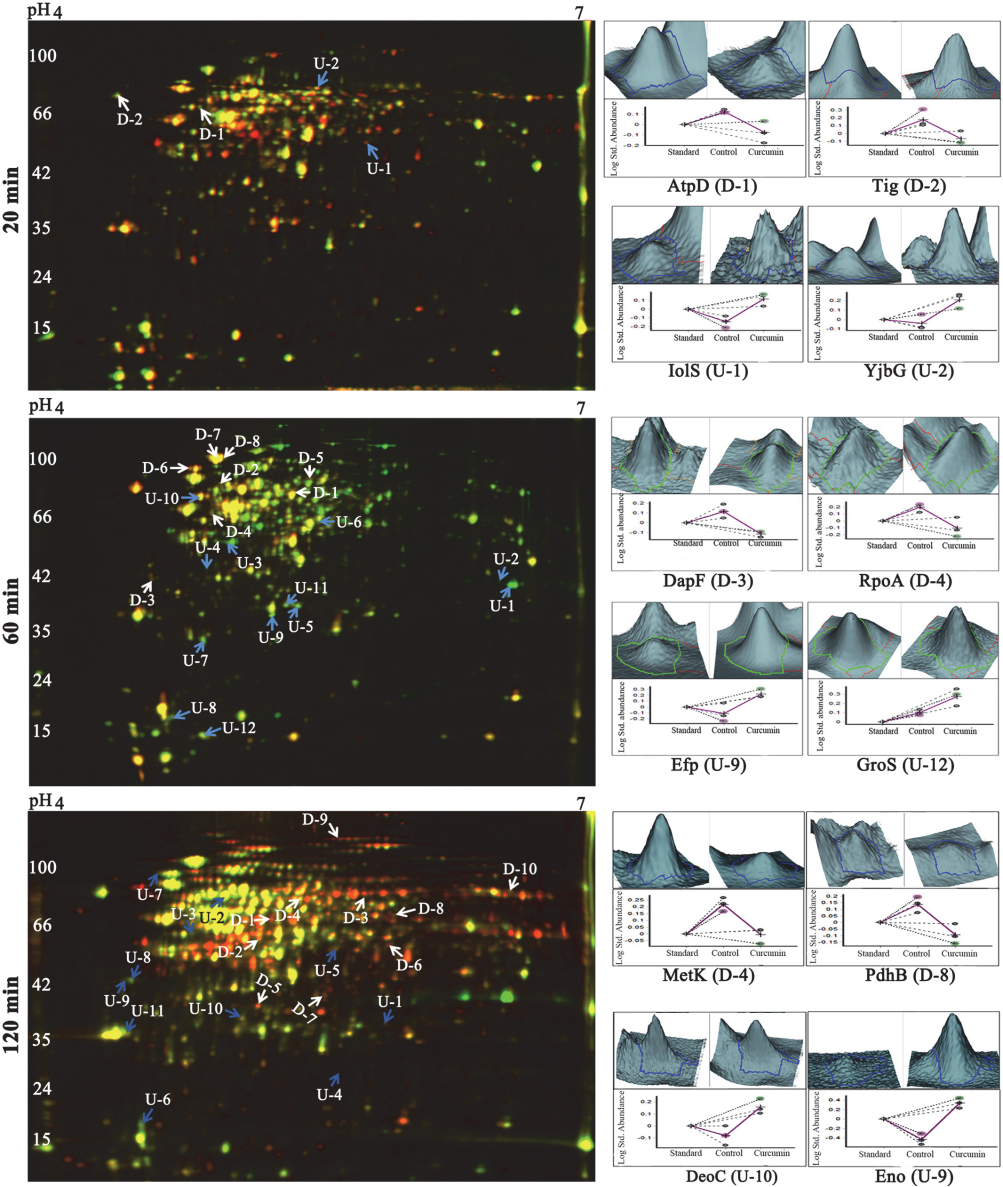
Temporal proteome changes of *B*. *subtilis* under curcumin treatment identified using 2D-DIGE. Representative overlapped (Cy3 and Cy5) 2D-DIGE images of *B*. *subtilis* proteome in response to the curcumin treatment (20, 60 and 120 min). 60 μg of each sample; control, curcumin treated (20, 60 and 120 min) and internal standard were labelled with Cy3, Cy5 and Cy2, respectively (Dye swapping was performed). Proteins were separated in first dimension IEF on 24 cm IPG strips of pH 4–7 range followed by second dimension separation on 12.5% SDS-PAGE. 3D view and BVA graphs of selected differentially expressed proteins (*p* < 0.05) identified from each time point of curcumin treatment in 2D-DIGE are shown. Data is represented as mean ± SE (where n = 3).

Comparative analysis of control and curcumin treated [after immediate (20 min), intermediate (60 min) and long treatment (120 min)] was performed by using 2D-DIGE, Nearly, 2000 protein spots were identified in DIA module of DeCyder software (GE Healthcare). Three sets of analysis i.e. control *vs*. 20 min, control *vs*. 60 min and control *vs*. 120 min were performed separately. Analysis of set-1 (control *vs*. 20 min treated) revealed differential expression of 4 protein spots with statistical significance (*p* ≤ 0.05); among which 2 spots showed up-regulation and 2 spots were down-regulated. Set-II analysis (control *vs*. 60 min) exhibited differential expression of 20 protein spots; among which 12 spots were up-regulated and 8 protein spots found to be down-regulated. While the analysis of set-III (control *vs*. 120 min treated) revealed differential expression of 21 protein spots, among which 10 spots were down-regulated and 11 spots were up-regulated. Representative combined DIGE image, 3D and BVA graph views of selected differentially expressed protein spots are displayed in the Fig 2 and S2 Fig. In 2D-DIGE analysis (DeCyder, GE Heathcare), only the statistically significant (*p ≤* 0.05; t-test and one way ANOVA) differentially expressed (fold-change > 1.5) proteins spots were considered for subsequent MALDI-TOF/TOF protein identification. All the protein spots showing differential expression in 2D-DIGE from all the three time points were excised from a preparative gel stained with CBB and subjected to MS identification. MALDI-TOF/TOF MS and MS/MS analysis successfully established the identity of 4 proteins in 20 min, 20 proteins in 60 min and 21 proteins in 120 min analysis (Table 1 & S1 Table). Among these differentially expressed proteins, ATP synthase subunit beta was common between 20 and 60 min and DNA-directed RNA polymerase alpha chain protein was common between 60 and 120 min.

**Table 1:** List of differentially expressed proteins in *B*. *subtilis* due to curcumin treatment obtained from DIGE analysis and its comparison with iTRAQ analysis^$^.

****Spot No****	****Uniprot ID****	****Name of the protein****	****Gene names****	****M.W (kDa)****	****No. of peptides****	****Fold change****	****iTRAQ (Orbitrap)****	****iTRAQ (QTOF)****	****ProteinScore****	****Ion Score****	****p-value****
**20 min curcumin treatment**											
D-1*	P37809	ATP synthase subunit beta	AtpD	51.42	19	-1.56	NS	NS	662	423	0.032
**D-2**	P80698	Trigger factor	Tig	47.48	11	-1.78	-1.21	-1.25	579	426	0.039
U-1*	P46336	Protein IolS	IolS	35.17	13	1.85	NS	2.39	167	104	0.01
**U-2**	O31605	Oligoendopeptidase F homolog	YjbG	77.07	4	1.78	1.59	1.58	109	85	0.019
**60 min curcumin treatment**											
D-1*	P37253	Ketol-acid reductoisomerase	IlvC	37.43	19	-1.67	NS	NI	1240	1163	0.043
D-2*	P42973	Aryl-phospho-beta-D-glucosidase BglA	BglA	54.84	18	-1.69	NS	NS	266	229	0.04
D-3 #	O32114	Diaminopimelate epimerase	DapF	30.85	4	-1.72	NI	NI	109	100	0.0067
D-4*	P20429	DNA-directed RNA polymerase alpha chain	RpoA	34.77	13	-1.95	NS	-1.42	245	200	0.029
D-5*	P22250	Glutamate—tRNA ligase	GltX	55.72	36	-2.23	NS	NS	783	648	0.026
**D-6**	Q07836	Uncharacterized protein yxxG	YxxG	16.43	4	-2.72	-1.61	-5.00	274	207	0.027
**D-7**	P80868	Elongation factor G	FusA	76.62	50	-5.01	-1.45	-1.48	1120	889	0.041
**D-8**	P80868	Elongation factor G	FusA	76.62	49	-5.92	-1.45	-1.48	1090	884	0.014
**U-1**	O32201	Protein LiaH	LiaH	25.69	31	17	6.63	2.23	788	663	0.0017
U-2*	P21464	30S ribosomal protein S2	RpsB	27.96	15	4.22	NS	-1.34	601	534	0.00026
**U-3**	P49814	Malate dehydrogenase	Mdh	33.64	13	2.97	2.17	1.63	628	586	0.047
U-4*	P80866	Vegetative protein 296	YurY	29.03	9	2.95	NS	NS	110	96	0.035
U-5*	P17820	Chaperone protein dnaK	DnaK	66	29	2.79	NS	NS	1000	793	0.0041
**U-6**	P54550	Probable NADH-dependent flavin oxidoreductase yqiM	YqiM	37.58	14	2.33	1.37	2.82	513	463	0.05
**U-7**	P80864	Probable thiol peroxidise	Tpx	18.31	10	2.18	1.56	1.52	303	233	0.0053
**U-8**	P96611	Thioredoxin-like protein ydbP	YdbP	12.43	5	2.11	1.39	1.39	120	102	0.032
U-9*	P49778	Elongation factor P	Efp	20.47	7	2.07	NS	NS	203	175	0.032
U-10*	P37809	ATP synthase beta chain	AtpD	51.42	29	1.96	NS	NS	1350	1192	0.045
**U-11**	O35022	FMN-dependent NADH-azoreductase 1	AzoR1	22.98	17	1.78	1.85	NI	436	391	0.036
**U-12**	P28599	10 kDa chaperonin	GroS	10.17	5	1.51	1.81	1.65	157	122	0.038
**120 min curcumin treatment**											
**D-1**	P23630	Diaminopimelate decarboxylase	LysA	48.7	18	-3.64	-1.35	-1.51	482	414	0.0034
**D-2**	P30950	Delta-aminolevulinic acid dehydratase	HemB	36.2	4	-3.57	-1.52	-1.33	146	102	0.012
**D-3**	P32395	Uroporphyrinogen decarboxylase	HemE	39.64	3	-3.57	-1.29	1.62	208	164	0.039
**D-4**	P54419	S-adenosylmethionine synthetase	MetK	44	10	-1.67	-2.33	-2.45	104	83	0.0079
D-5*	P39121	Deoxyribose-phosphate aldolase	DeoC	22.19	6	-2.35	NS	NS	175	150	0.0045
**D-6**	P05654	Aspartate carbamoyltransferase	PyrB	34.31	16	-2.29	-1.94	NI	468	373	0.028
**D-7**	P52996	3-methyl-2-oxobutanoate hydroxymethyltransferase	PanB	30	3	-1.94	NS	-1.92	328	284	0.037
D-8*	P21882	Pyruvate dehydrogenase E1 component subunit beta	PdhB	35.47	26	-1.69	NS	NS	317	171	0.015
D-9*	Q9KWU4	Pyruvate carboxylase	Pyc	127.9	30	-2.75	NS	NS	315	188	0.0012
**D-10**	O32117	NADH dehydrogenase-like protein yutJ	YutJ	39.75	19	-2.28	-1.51	NI	865	737	0.014
**U-1**	P80244	ATP-dependent Clp protease proteolytic subunit	ClpP	21.6	5	1.63	2.26	2.35	323	241	0.05
U-2*	P21880	Dihydrolipoyl dehydrogenase	PdhD	49.7	20	1.67	NS	-1.24	996	929	0.024
U-3*	P20429	DNA-directed RNA polymerase alpha chain	RpoA	34.77	13	2	NS	-1.28	245	200	0.029
**U-4**	P81101	Ribosome recycling factor	Frr	20.62	10	2.56	1.50	1.56	346	273	0.047
**U-5**	O32259	Lactate utilization protein C	LutC	26.27	6	2.58	1.26	NS	223	199	0.02
U-6*	P37487	Manganese-dependent inorganic pyrophosphatase	PpaC	34	4	2.6	NS	NS	379	283	0.04
**U-7**	P28598	60 kDa chaperonin	GroL	57.25	25	2.84	2.10	1.95	1340	1224	0.048
**U-8**	P15874	GrpE protein (HSP-70 cofactor)	GrpE	21.53	6	3.98	1.50	NS	186	167	0.023
U-9*	P37869	Enolase	Eno	46.42	8	5.86	NS	NS	75	49	0.0034
U-10*	P39121	Deoxyribose-phosphate aldolase	DeoC	23.47	13	1.72	NS	NS	611	524	0.041
**U-11**	P09339	Aconitate hydratase	CitB	99.61	28	4.13	4.56	3.88	789	664	0.0015

$ This is a partial list having selected candidates with >1.5 fold change and complete list is provided in S2 Table

#: Proteins unique in DIGE, Bold: Same trend in both DIGE and iTRAQ (Orbitrap data);

* or NS: No significant change in iTRAQ in Orbitrap data (less than 1.2 fold up and down);

NI- Not identified in iTRAQ analysis.

### Effect of Curcumin Treatment on *B*. *subtilis* Proteome Revealed by iTRAQ-based Quantitative Proteomics Analysis

**Fig 3:**
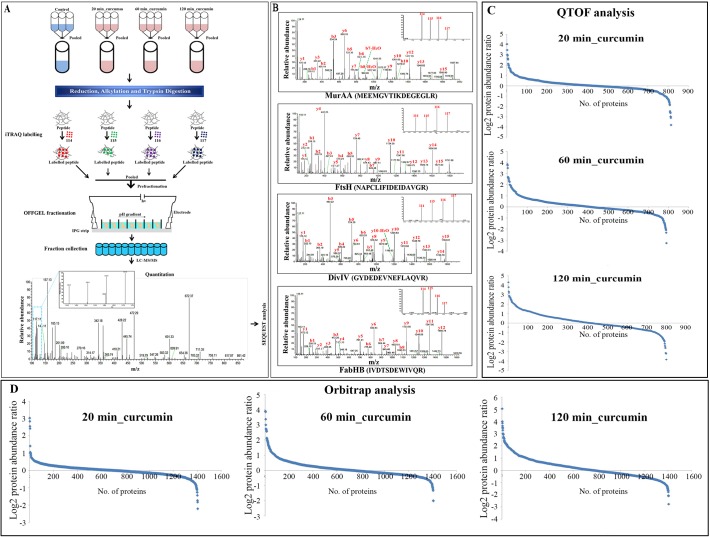
Schematic representation of experimental strategy for temporal proteome analysis of *B*. *subtilis* under curcumin treatment by iTRAQ-based quantitative proteomics. **(A)** Samples processed in triplicate were pooled from control, 20, 60 and 120 min curcumin treated cultures and labelled with iTRAQ reagent 114, 115, 116 and 117, respectively. The labelled peptides were fractionated in OFFGEL fractionators using high resolution (24 cm; 3–10 pH) IPG strips and each fraction was desalted using C18 tips. Desalted fractions were subjected to LTQ-Orbitrap Velos mass spectrometer for protein identification and quantitation. **(B)** Representative MS/MS spectrum of a few selected differentially expressed proteins identified after curcumin treatment. UDP-N-acetylglucosamine 1-carboxyvinyltransferase 1 (MurAA), ATP-dependent zinc metalloprotease FtsH, Septum site-determining protein (DivIVA), and 3-oxoacyl-[acyl-carrier-protein] synthase 3 protein 1 (FabHB). Inset showing the iTRAQ reporter ion intensities for representative peptides in control and curcumin treated samples. **(C)** S-curve analysis exhibiting distribution of the differentially expressed proteins in *B*. *subtilis* after 20, 60 and 120 min of curcumin treatment identified using Q-TOF (average of three triplicate runs). **(D)** S-curve analysis exhibiting distribution of the differentially expressed proteins in 20, 60 and 120 min curcumin treated *B*. *subtilis* identified using LTQ-orbitrap.

iTRAQ-based quantitative proteomics analysis at all three time points of curcumin treated and control *B*. *subtilis* using Q-TOF revealed the identification of 864 proteins in technical triplicates whereas 1414 proteins were identified using LTQ- Orbitrap Velos mass spectrometer (Fig 3A and 3B). In case of iTRAQ-based quantitative proteomic analysis, only proteins with 1% FDR were considered for analysis. Calculation of FDR is important to ensure the validity of results. Proteome Discoverer workbench allows automated calculation of false discovery rate. Percolator (component of Proteome Discoverer 1.3) improves the sensitivity of existing database search algorithms at a constant false discovery rate. Target FDR (strict), which specifies a strict target false discovery rate for peptide matches with high confidence, was employed for analysis. Quality of the iTRAQ data was checked by S-curve analysis of QTOF and Orbitrap data. QTOF analysis indicated differential expression of 20%, 26% and 40% the total proteome; while the Orbitrap analysis exhibited 6%, 17% and 40% alterations in *B*. *subtilis* proteome in 20 min, 60 min and 120 min curcumin treatment, respectively with 1.5-fold change (Fig 3C and 3D and S3 Fig). The complete list of the identified proteins from LTQ-Orbitrap and Q-TOF along with the peptide sequence, sequence coverage, unique peptides, PSM, modifications, X Corr and iTRAQ ratios information is provided in supplementary information (S2 Table). The comparative quantitative proteomic analysis between QTOF and Orbitrap indicated differential expression (1.5 fold change) of 15 proteins in 20 min (8 up-regulated and 7 down-regulated), 81 proteins in 60 min (69 up-regulated and 12 down-regulated) and 210 proteins in 120 min (154 up- regulated and 56 down-regulated) of curcumin treatment with similar trend in both of the mass-spectrometric analysis (S2 Table). In addition, we have compared the Orbitrap and QTOF data without any fold-change criteria. In case of Orbitrap, we identified 1414 proteins whereas in case of QTOF, we identified 864 proteins. The coverage in QTOF analysis is quite low compared to Orbitrap analysis. In case of 20 min, 273 up-regulated proteins (39% in Orbitrap data and 63% in QTOF data) and 229 down-regulated proteins (33% of Orbitrap data and 60% of QTOF data) exhibited similar trend. In case of 60 min, 288 up-regulated proteins (39% of Orbitrap data and 69% of QTOF data) and 246 down-regulated proteins (37% of Orbitrap data and 66% of QTOF data) showed similar trend of differential expression. In case of 120 min, 332 up-regulated proteins (43% of Orbitrap data and 80% of QTOF data) and 286 down-regulated proteins (45% in Orbitrap data and 75% in QTOF data) were found to be of similar trends. The low percentage of similarity in Orbitrap data is due to the low coverage in QTOF (S4A Fig). More than 90% proteins identified in QTOF were also identified in Orbitrap analysis (S4B Fig). Few proteins such as 3-oxoacyl-[acyl-carrier-protein] synthase 3 protein 2, ATP-dependent Clp protease proteolytic subunit and 60 kDa chaperonin exhibited similar trend across the time points in both Q-TOF and LTQ-orbitrap analysis (Fig 3B and S2 Table).

In addition, comparative analysis of the findings obtained from the two complementary techniques indicated that most of the differentially expressed protein identified in DIGE were also detected in iTRAQ analysis (Orbitrap data), except diaminopimelate epimerase, differential expression of which was only identified in DIGE (S5A and S5B Fig). Further, the comparison among the time point analysis revealed that quite a few differential expressed proteins were time point specific (S6 Fig). The MS/MS spectra of a few selected proteins along with the inset showing the iTRAQ reporter ion intensities for representative peptides in control and curcumin treated (20, 60 and 120 min) samples are depicted in the Fig 3B.

### Alteration of Protein Clusters after Curcumin Treatment

**Fig 4:**
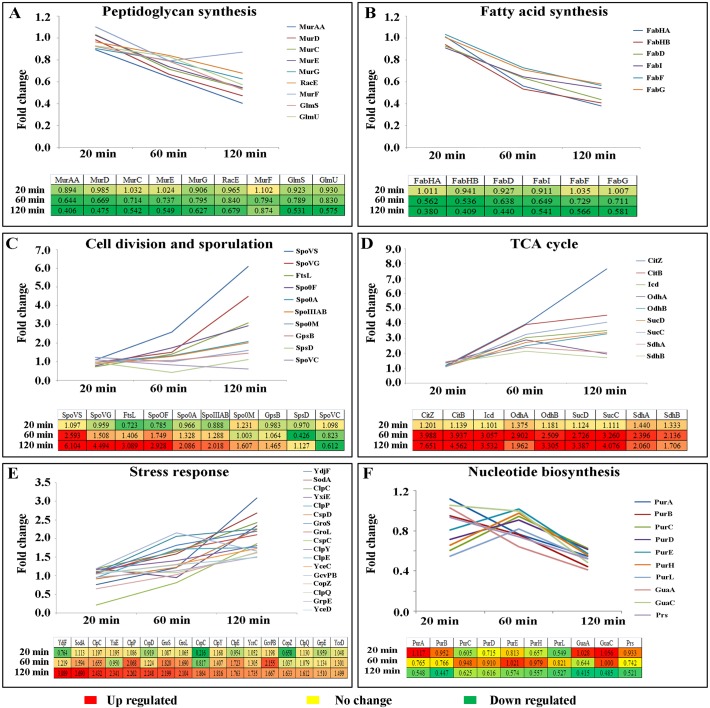
Quantitative profiles of the differentially expressed proteins involved in diverse biological processes identified in iTRAQ-based quantitative proteomics analysis using LTQ-Orbitrap. **(A)** Peptidoglycan biosynthesis, **(B)** Fatty acid synthesis, **(C)** Cell division and sporulation, **(D)** TCA cycle, **(E)** Stress response and **(F)** Nucleotide biosynthesis. Data from QTOF is provided in the S2 Fig.

The global proteome analysis using both DIGE and iTRAQ characterized the physiological responses of *B*. *subtilis* to curcumin treatment. Expectedly, the differential proteomic analysis highlighted the induction of a cluster of proteins involved in stress response; additionally altered expression levels of proteins associated with cell division, sporulation and central metabolism. The universal chaperone proteins such as GroEL and GroES showed induction as time of curcumin treatment increased. Chaperone-proteases required for protein folding and degradation of aggregated proteins were also induced linearly with exposure time. Similarly, quite a few cell division/sporulation and central metabolism proteins were also induced with negligible change after 20 min but showed linear induction at 60 and 120 min treatment. Proteins associated with nucleotide biosynthesis showed repression with respect to the exposure time. In addition, quite a few proteins involved in peptidoglycan synthesis and fatty acid synthesis exhibited linear repression in expression level with respect to curcumin treatment. The quantitation of proteins involved in fatty acid synthesis, cell wall biogenesis, cell division, sporulation, TCA cycle, nucleotide biosynthesis and stress physiology are summarized in the Table 2, Fig 4 and S7 Fig.

**Table 2:** Partial list of differentially expressed proteins in *B*. *subtilis* due to curcumin treatment obtained from iTRAQ analysis*.

UniProt ID	Name of the protein	Gene name	Coverage	Uni. Peptides (Orbitrap)	Fold change (Orbitrap)			Uni. Peptides (Q-TOF)	Fold change (QTOF)		
					20 min	60 min	120 min		20 min	60 min	120 min
**Cell wall synthesis**											
P70965	UDP-N-acetylglucosamine 1-carboxyvinyltransferase 1	MurAA	35.32	13	0.89	0.64	0.41	13	1.36	0.702	0.549
Q03522	UDP-N-acetylmuramoylalanine—D-glutamate ligase	MurD	31.04	13	0.98	0.67	0.47	4	1.115	1.039	0.812
P40778	UDP-N-acetylmuramate—L-alanine ligase	MurC	25.46	9	1.03	0.71	0.54	6	1.296	1.170	0.580
Q03523	UDP-N-acetylmuramoyl-L-alanyl-D-glutamate—2,6-diaminopimelate ligase	MurE	37.04	14	1.02	0.74	0.55	9	0.840	0.773	0.379
P37585	UDP-N-acetylglucosamine—N-acetylmuramyl-(pentapeptide) pyrophosphoryl-undecaprenol N-acetylglucosamine transferase	MurG	37.47	11	0.91	0.8	0.63	3	0.693	0.682	0.462
P94556	Glutamate racemase 1	RacE	25.37	4	0.97	0.84	0.68	2	0.773	0.857	0.539
P96613	UDP-N-acetylmuramoyl-tripeptide—D-alanyl-D-alanine ligase	MurF	30.20	8	1.10	0.79	0.87	3	1.721	0.934	0.846
P0CI73	Glutamine—fructose-6-phosphate aminotransferase [isomerizing]	GlmS	30.33	15	0.92	0.79	0.53	10	0.891	0.761	0.535
P14192	Bifunctional protein GlmU	GlmU	23.03	8	0.93	0.83	0.58	7	2.139	1.505	0.885
**Cell division and Sporulation**											
P45693	Stage V sporulation protein S	SpoVS	38.37	2	1.10	2.59	6.10	3	3.703	4.052	6.623
P28015	Putative septation protein SpoVG	SpoVG	42.27	4	0.96	1.51	4.49	2	0.912	0.712	2.091
Q07867	Cell division protein FtsL	FtsL	8.55	1	0.72	1.41	3.09	NI	NI	NI	NI
P0CI74	Cell cycle protein GpsB	GpsB	43.88	4	0.98	1.06	1.46	2	1.173	1.452	1.772
P06628	Sporulation initiation phosphotransferase F	SpoOF	23.39	2	0.79	1.75	2.93	2	0.566	1.686	2.371
Q01368	Stage III sporulation protein AB	SpoIIIAB	5.26	1	0.89	1.29	2.02	NI	NI	NI	NI
P06534	Stage 0 sporulation protein A	SpoOA	13.86	3	0.97	1.33	2.09	1			1.158
P71088	Sporulation-control protein spo0M	SpoOM	37.21	10	1.23	1.00	1.61	5	1.446	0.986	0.877
P39624	Spore coat polysaccharide biosynthesis protein SpsD	SpsD	4.84	1	0.97	0.43	1.13	NI	NI	NI	NI
P37470	Peptidyl-tRNA hydrolase	SpoVC	13.83	2	1.10	0.82	0.61	2	2.023	0.587	0.877
**Fatty acid synthesis**											
O34746	3-oxoacyl-[acyl-carrier-protein] synthase 3 protein 1	FabHA	45.83	13	1.01	0.56	0.38	6	0.914	0.584	0.438
O07600	3-oxoacyl-[acyl-carrier-protein] synthase 3 protein 2	FabHB	18.15	6	0.94	0.54	0.41	2	1.079	0.407	0.480
P71019	Malonyl CoA-acyl carrier protein transacylase	FabD	48.26	14	0.93	0.64	0.44	13	0.976	0.620	0.564
P54616	Enoyl-[acyl-carrier-protein] reductase [NADH] FabI	FabI	51.16	11	0.91	0.65	0.54	6	0.975	0.905	0.787
O34340	3-oxoacyl-[acyl-carrier-protein] synthase 2	FabF	48.67	14	1.03	0.73	0.57	11	1.146	0.858	0.818
P51831	3-oxoacyl-[acyl-carrier-protein] reductase FabG	FabG	61.79	12	1.01	0.71	0.58	7	1.318	0.912	0.794
**Stress response**											
P37571	Negative regulator of genetic competence ClpC/MecB	ClpC	59.88	45	1.20	1.65	2.43	29	1.264	1.809	2.746
P80244	ATP-dependent Clp protease proteolytic subunit	ClpP	43.15	8	1.09	2.07	2.26	4	1.283	2.136	2.208
P39778	ATP-dependent protease ATPase subunit ClpY	ClpY	35.55	14	1.17	1.41	1.82	5	1.835	1.589	2.231
O31673	ATP-dependent Clp protease ATP-binding subunit ClpE	ClpE	16.17	5	0.95	1.72	1.76	NI	NI	NI	NI
P39070	ATP-dependent protease subunit ClpQ	ClpQ	21.55	4	1.13	1.08	1.61	NI	NI	NI	NI
P54617	Phage shock protein A homolog	YdjF	58.59	11	0.76	1.22	3.09	6	1.065	1.472	2.324
P54375	Superoxide dismutase [Mn]	SodA	67.82	9	1.11	1.59	2.69	6	1.148	1.224	2.161
P42297	Universal stress protein YxiE	YxiE	45.27	5	1.19	0.95	2.34	3	0.901	0.859	1.609
P51777	Cold shock protein CspD	CspD	89.39	5	0.92	1.22	2.25	3	0.920	1.452	1.325
P28599	10 kDa chaperonin	GroS	73.40	7	1.09	1.82	2.20	5	1.329	1.824	2.662
P28598	60 kDa chaperonin	GroL	74.63	39	1.07	1.69	2.10	24	1.187	1.885	2.289
P39158	Cold shock protein CspC	CspC	59.09	5	0.22	0.82	1.86	1	0.304	0.863	1.842
P81100	Stress response protein SCP2	YceC	37.69	6	1.05	1.30	1.74	2	1.381	1.308	2.002
P54377	Probable glycine dehydrogenase [decarboxylating] subunit 2	GcvPB	9.43	3	1.20	2.15	1.67	1	0.693	2.848	2.690
O32221	Copper chaperone CopZ	CopZ	68.12	3	0.66	1.04	1.63	1		1.427	0.873
P15874	Protein GrpE	GrpE	57.75	10	0.96	1.13	1.51	5	1.399	1.455	1.497
P80875	General stress protein 16U	YceD	49.74	6	1.05	1.30	1.50	6	1.085	1.542	1.672
**TCA cycle**											
P39120	Citrate synthase 2	CitZ	31.18	11	1.20	3.99	7.65	7	1.621	3.706	8.131
P09339	Aconitate hydratase	CitB	33.22	25	1.14	3.94	4.56	12	2.032	4.297	4.724
P39126	Isocitrate dehydrogenase [NADP]	Icd	44.21	20	1.10	3.06	3.53	9	2.008	2.694	3.139
P23129	2-oxoglutarate dehydrogenase E1 component	OdhA	35.28	27	1.37	2.90	1.96	14	1.286	2.223	1.744
P16263	Dihydrolipoyllysine-residue succinyltransferase component of 2-oxoglutarate dehydrogenase complex	OdhB	54.44	18	1.18	2.51	3.30	13	2.203	4.063	3.053
P80865	Succinyl-CoA ligase [ADP-forming] subunit alpha	SucD	33.00	6	1.12	2.73	3.39	4	1.553	2.023	3.060
P80886	Succinyl-CoA ligase [ADP-forming] subunit beta	SucC	55.32	22	1.11	3.26	4.08	15	1.252	5.002	4.043
P08065	Succinate dehydrogenase flavoprotein subunit	SdhA	49.32	22	1.44	2.40	2.06	9	1.061	2.320	2.220
P08066	Succinate dehydrogenase iron-sulfur subunit	SdhB	27.27	6	1.33	2.14	1.71	3	1.347	2.216	1.813
**Nucleotide biosynthesis**											
P29726	Adenylosuccinate synthetase	PurA	26.05	10	1.12	0.76	0.55	7	1.189	0.814	1.119
P12047	Adenylosuccinate lyase	PurB	35.27	15	0.95	0.77	0.45	4	0.966	0.722	0.535
P12046	Phosphoribosylaminoimidazole-succinocarboxamide synthase	PurC	4.98	1	0.61	0.95	0.62	NI	NI	NI	NI
P12039	Phosphoribosylamine—glycine ligase	PurD	12.56	4	0.72	0.91	0.62	NI	NI	NI	NI
P12044	N5-carboxyaminoimidazole ribonucleotide mutase	PurE	19.75	2	0.81	1.02	0.57	2	0.740	0.761	1.417
P12048	Bifunctional purine biosynthesis protein PurH	PurH	27.54	11	0.66	0.98	0.56	5	0.491	1.212	0.584
P12042	Phosphoribosylformylglycinamidine synthase 2	PurL	3.10	2	0.55	0.82	0.53	NI	NI	NI	NI
P29727	GMP synthase [glutamine-hydrolyzing]	GuaA	58.67	27	1.03	0.64	0.42	17	1.162	0.714	0.538
O05269	GMP reductase	GuaC	48.16	10	1.06	1.00	0.49	4	1.766	2.612	1.020
P14193	Ribose-phosphate pyrophosphokinase	Prs	35.65	11	0.93	0.74	0.52	8	0.889	0.884	0.707

* This is a partial list having selected candidates; complete list is provided in S2 Table

NI- Not identified in Q-TOF

### Modulation of Biological Process and Physiological Pathways in *B*. *subtilis* due to Curcumin Treatment

KOBAS and DAVID analysis performed with the differentially expressed proteins (1% FDR and 1.5 fold-change) indicated immediate exposure (20 min treatment) of the drug did not affect the physiological pathway significantly. Intermediate exposure (60 min treatment) of *B*. *subtilis* to curcumin caused the alterations of TCA cycle, glyoxylate metabolism, anaerobic respiration, gluconeogenesis and glycine cleavage (S3 Table). Prolonged exposure of curcumin (120 min) effectively altered the fatty acid synthesis, peptidoglycan synthesis, anaerobic respiration, TCA cycle and propionate metabolism (S3 Table).

### Effect of Curcumin on Respiratory Activity, Ion Leakage and Metabolic Activity

**Fig 5:**
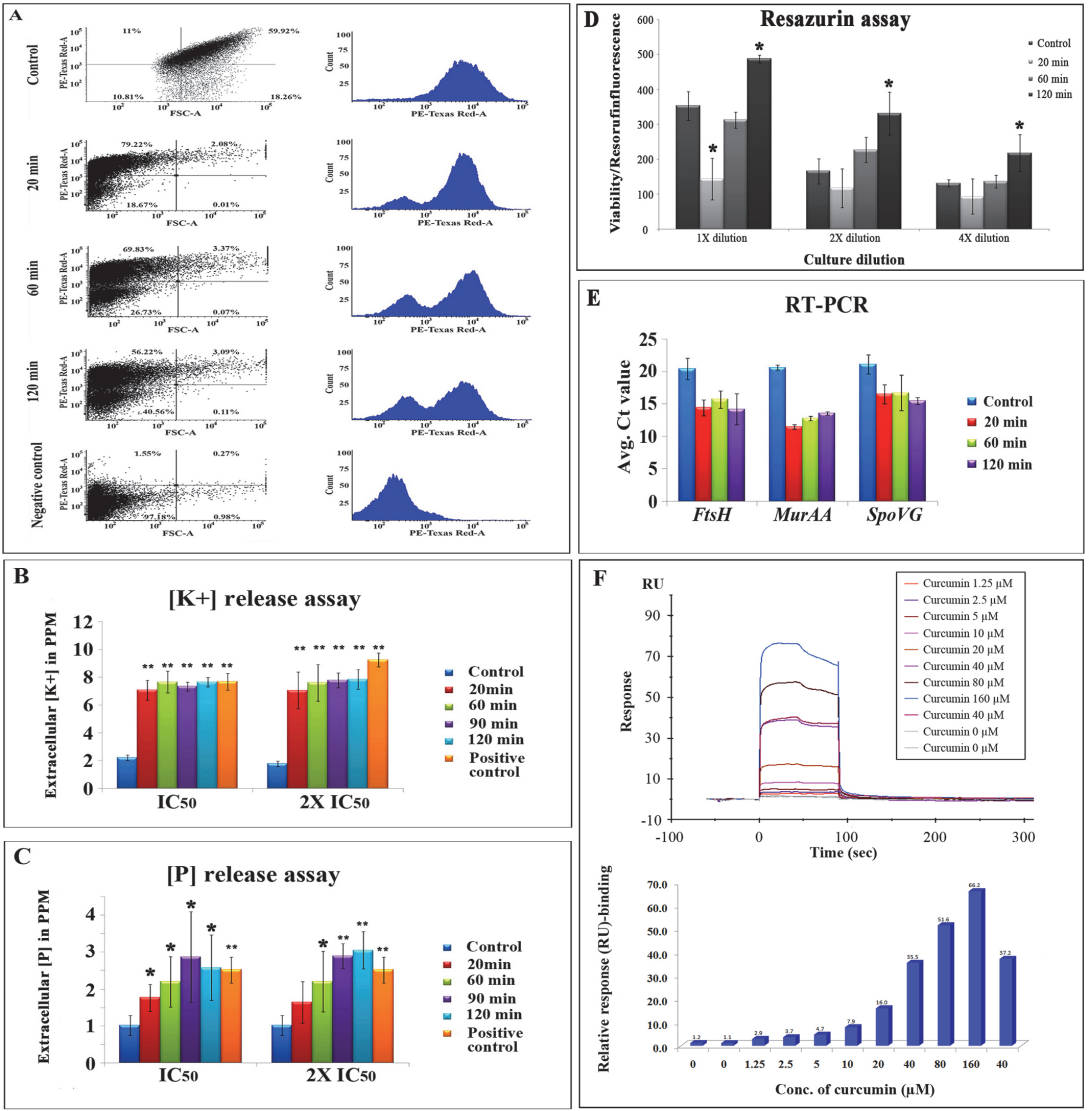
(A) CTC staining and flow cytometric analysis for respiratory activity. Graphical representation of CTC mean intensity (PE-Texas Red-A) *vs*. FSC-A obtained in the FACS analysis of control, 20, 60 and 120 min curcumin treated samples and negative control. Both dot plot and histogram representations are displayed for each sample. **(B) & (C)** Potassium and phosphorus leakage assay; curcumin (20 and 40 μM) was added to the *B*. *subtilis* in HEPES-glucose medium and K^+^ and P levels were measured at 20, 60, 90 and 120 min time intervals, and also in the untreated control and positive control (heated at 70° C for 30 min) samples using ICP-AES and data was normalized with baseline HEPES-glucose medium. **(D)** Metabolic activity assay using resazurin. 20 min curcumin treatment has showed lower metabolic activity whereas the metabolic activity increased as time of exposure increased to 60 min and 120 min as compared to control. * indicates *p* < 0.05. **(E)** Gene expression analysis using RT-PCR for *murAA*, *spoVG* and *ftsH* genes and the relative expression was calculated by taking mean C_t_ values from triplicate runs. * indicates *p* < 0.05 and ** indicates *p* < 0.001. **(F)** Physical interaction analysis of curumin with *B*. *subtilis* FtsZ immobilized on CM-5 sensor chip. The interaction was monitored by measuring the response unit and the response unit was increased as the concentration of curcumin increased. Both sensorgram and the bar diagram showing the binding to FtsZ was displayed.

Respiratory activity of the drug treated cells were analyzed by CTC (5-cyano-2, 3-ditolyl tetrazolium chloride) staining and flow cytometric analysis. CTC is a non-fluorescent dye which is converted into red-colored fluorescent formazan in respiratory active cells. Control and curcumin treated (20 min, 60 min and 120 min) samples were dual stained with 5 mM CTC for 30 min at 37° C in dark followed by counter staining with DAPI. The total population of viable cells were determined by measuring the DAPI with UV laser (358 nm) and the CTC stained cells were measured by exciting with red florescence laser (630 nm) in FACS. Both forward and side scattering were measured to analyze the size of the cell and to remove the contaminants respectively. Curcumin treatment cells showed reduced formazan florescence with respect to the untreated control cells, and the reduction in formazan florescence found to be increased with the time of drug exposure. A negative control was prepared by disturbing the membrane using 2.8% formaldehyde and 0.04% glutaraldehyde for 30 min prior to the CTC staining, for which the CTC intensity was almost zero (Fig 5A).

The extracellular inorganic phosphate and potassium levels were monitored at different time intervals of curcumin exposure using ICP-AES. The extracellular K^+^ and P levels of curcumin treated cells (both IC_50_ and 2 x IC_50_) found to be increased with respect to the untreated control cells. Nearly three times increase in the extracellular K^+^ leakage was observed after 20 min exposure of curcumin and the levels were remained unaltered with respect to the untreated control as time of curcumin exposure increased to 120 min. Moreover, inorganic phosphate levels were found to be increased with the increase in exposure time of curcumin from 20 min to 120 min. In case of positive control (the cells were heated at 70° C for 30 min), the extracellular K^+^ and P levels were found to be higher compared to the untreated control; the similar trend as exhibited by the curcumin treated samples. Two different concentrations of curcumin were used to monitor extracellular K^+^ and P levels, but no significant difference was observed between the IC_50_ and 2 x IC_50_ concentrations of the drug (Fig 5B and 5C).

Non-fluorescent resazurin blue dye is converted into pink resorufin by the active metabolic enzymes from central metabolism in the viable cells. *B*. *sublitis* cells treated with curcumin exhibited reduced metabolic activity at the immediate exposure (20 min) of curcumin whereas the metabolic activity was found to be increased at the intermediate (60 min) and long exposure (120 min) of the drug as compared to untreated control cells (Fig 5D).

### Gene Expression Analysis using RT-PCR and SPR Interaction Analysis

The quantitative gene expression analysis of three selected candidates including *murAA* (UDP-N-acetylglucosamine 1-carboxyvinyltransferase 2), *spoVG* (putative septation protein SpoVG) and *ftsH* (ATP-dependent zinc metalloprotease FtsH) associated with cell division was performed using RT-PCR analysis. Gene expression analysis of *ftsH and spoVG* showed significant elevation in their expression at transcriptome levels, which is consistence with the proteomic data; whereas *murAA* exhibited elevated expression at mRNA level after curcumin treatment, but showed reverse trend of differential expression in proteomic analysis (Fig 5E).

The SPR-biosensor based interaction analysis has showed very good binding of curcumin with FtsZ and the interaction was measured by calculating the response unit (RU). Eight different concentrations of curcumin was used for interaction analysis and all the concentrations showed binding of curcumin to the FtsZ in concentration-dependent manner i.e. as the concentration of curcumin increases, the observed response unit was also increased linearly. The duplicates (40 μM) have showed similar response with FtsZ, which indicates the good quality of the data (Fig 5F).

## Discussion

Differential proteomic analysis is found to be very effective for comprehensive analysis of microbial responses to different environmental stress conditions and drug treatments. Previous studies have demonstrated anti-tumergenic, anti-inflammatory, anti-mutagenic and anti-oxidant activities of curcumin [3,5]. Although, quite a few earlier studies have investigated the effect of curcumin on *B*. *subtilis*, the mechanism of action of the drug and its role in filamentation as well as exact molecular targets are still unclear and proteome level analysis has not been performed hitherto. To the best of our knowledge, we report here the first comprehensive proteomic analysis describing the effect of curcumin on *B*. *subtilis* proteome to understand the mode of action of curcumin and its primary cellular targets. The first and foremost comprehensive proteome mapping of *B*. *subtilis* was reported by Eymann et al. using classical 2-DE and obtained coverage was 745 proteins [17]. Later Wolff et al. have improved the coverage to 1218 proteins using a combination of 2-DE and iTRAQ [18]. In the present study, we were able to cover 1466 proteins using both DIGE and iTRAQ-based quantitative proteomics, which covered almost 60% of the entire vegetative proteome of *B*. *subtilis*. The present study demonstrates the application of temporal quantitative proteome analysis to understand the mechanism of action and possible targets of curcumin in *B*. *subtilis*. The altered proteins were found to be majorly involved in bacterial cell division, cell wall biosynthesis, fatty acid synthesis and central metabolism. In addition, the universal chaperone system (GroEL) required for FtsZ folding and the major protease (Clp family) system target FtsZ for degradation has been altered. We would also like to mention that this is the foremost global study contributing to understand the mechanism of action and putative targets of curcumin in *B*. *subtilis*.

**Fig 6:**
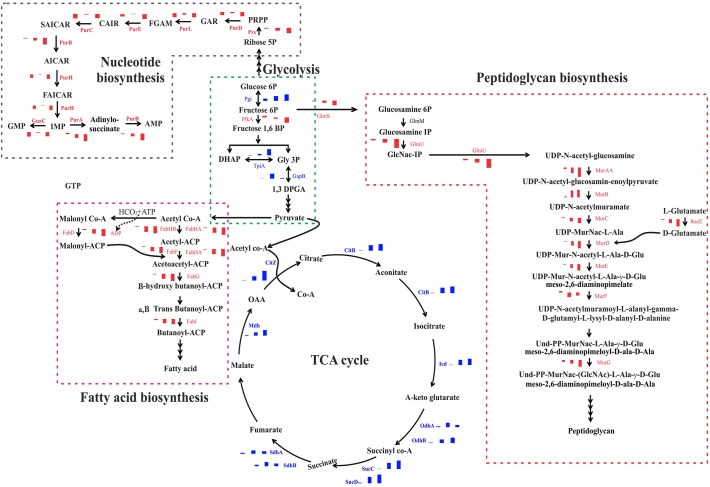
Modulation of essential physiological pathways in *B*. *subtilis* due to curcumin treatment. The pathway was built based on DAVID, KEGG and KOBAS analysis and the combined pathway was built manually. Red bar or name indicates down-regulation and blue bar and name indicates up-regulation. The three bars indicate the protein expression at 20, 60 and 120 min of curcumin treatment (expression levels obtained from LTQ-Orbitrap analysis). PRPP-5-Phospho-alpha-D-ribose 1-diphosphate; GAR-5'-Phosphoribosylglycinamide; FGAM-5'-Phosphoribosyl-N-formylglycinamidine; CAIR-5'-Phosphoribosyl-5-amino-4-imidazolecarboxylate; SAICAR-5'-Phosphoribosyl-4-(N-succinocarboxamide)-5-aminoimidazole; AICAR-5-Phosphoribosyl-4-carbamoyl-5-aminoimidazole; FAICAR-5'-Phosphoribosyl-5-formamido-4-imidazolecarboxamide; IMP-Inosine monophosphate.

Our proteomic analysis revealed modulation of quite a few members of cell wall biosynthesis and cell division machinery proteins. Peptidoglycan cross-linking is the major scaffold in cell wall to provide the strength and maintain the cell shape along with shape determining proteins [19]. Cell wall and cell division proteins express together to correlate the division with cell wall synthesis which reside in the division cell wall (DCW) cluster. Quite a few cell wall synthesizing proteins of DCW cluster were found to be repressed at the intermediate and long exposure of curcumin, whereas cell division proteins were slightly induced indicating that individual promoters up-stream to the each gene are affected, but the common promoter of DCW cluster probably remained unaffected [20]. MurAA, which catalyzes the first step in peptidoglycan synthesis and is linked to the rest of the Mur proteins involved in successive steps and also coordinate in cell division, was found to be repressed after curcumin treatment (Fig 6). Even though, MurAA protein level was repressed under curcumin treatment, its mRNA level expression was found to be elevated (Fig 5E). In order to find out the rationale behind this observation, expression levels of different proteins which have direct or indirect influence on MurAA expression at the protein level were analyzed carefully. Interestingly, we identified elevated level of ClpCP, which is a proteolytic enzyme causing degradation of MurAA during the 60 and 120 min of curcumin exposure [20]. In addition, GlmS and GlmU, two crucial enzymes required for cell wall synthesis have been found to be repressed; GlmS is also a target of ClpC [14] and its down-regulation may be a consequence of elevated cellular level of ClpCP. Additionally, cell wall stress specific marker protein like protein LiaH was also found to be induced under curcumin treatment; probably to protect the survival of the bacteria despite hampered cell wall biosynthesis under the stress condition. Induction of YdjF and YtrB proteins, which are marker proteins of cell wall damage and help in stabilizing the cell envelop, was also observed [21]. Interestingly, MreBH, MreB and Mbi, the major proteins involved in cell morphogenesis, were also found to be repressed at the late stages of curcumin treatment. Earlier studies have demonstrated that MreB knockouts strains and reduced expression of MreHB and Mbi lead to the filamentous cell morphology [22,23]. Further, FtsL-DivIB and MinJ proteins, which play an important role in the late stage vegetative septum and asymmetric septum formation during sporulation [24–27] were slightly induced due to the curcumin treatment. Further, ATP-dependent zinc metal protease (FtsH) that degrades the peptidoglycan layer and maintains lipid homeostasis during vegetative and sporulation was slightly induced at intermediate and late exposure. Gene expression analysis of FtsH also showed similar trend of induction at the transcription level probably to enhance the asymmetric division [28]. Interestingly, the SpoVG, asymmetric division regulator proteins were induced both at protein and transcriptome levels [29]. The induced late cell division proteins and sporulation proteins indicate the asymmetric division as an alternative of vegetative septum. Besides, Rai et al. showed that curcumin treatment severally perturbs the FtsZ polymerization dynamics required for the cell division and leads to filamentation. The biosensor-based interaction analysis has also showed binding of curcumin with FtsZ at the molecular level and may halt the function of polymerization (Fig 5F). Therefore, we anticipate that apart from disturbing the FtsZ polymerization, curcumin also affects the expression of cell division accessory proteins [12].

Since diverse classes of proteins associated with cell division and cell wall synthesis process were found to be deregulated due to the curcumin treatment, we were interested to verify the expression levels of molecular chaperones, which support the folding of the proteins. Clp proteins are highly conserved protease system in eubacteria and constitute ClpC, ClpX, ClpE, ClpY, ClpQ and ClpP in *B*. *subtilis* [30]. These proteins are generally induced under stress conditions and recognize the unfolded proteins, while ClpP is the proteolytic core, which degrades the unfolded proteins [31]. The increased expression levels of the multiple members of Clp family under curcumin treatment indicate aggregations or unfolding of the proteins in the cell. Additionally, Clp family has important role in cell division because FtsZ is one of the prime targets for ClpP, sporulation, motility and other physiological processes [32]. Similar results were reported previously indicating an increased expression of ClpP causes the degradation of FtsZ through its proteolytic activity and consequently promotes cell elongation [31]. We anticipate that the elevated levels of Clp family can target the cell division proteins and promote the elongation. However, GroEL and GroES, one of the major chaperon and co-chaperon systems in *B*. *subtilis* were found to be elevated due to curcumin treatment. GroL is a 60 kDa chaperonin system and GroS is a 10 kDa protein, which plays a fundamental role in protein folding and is considered to be highly conserved among eubacteria. A previous study by Kerner et al., demonstrated that GroEL system has more than 85 substrates involved in various physiological pathways in *E*. *coli* [33]. GroEL system is also involved in folding of cell division proteins such as FtsZ, FtsA, FtsI and FtsE proteins [34]. Increased level of GroEL might be due to the mis-folding of the essential cell division proteins under curcumin treatment leading to an urgent need for the excess of chaperone to repair the system. Moreover, GroEL is localized at the centre of the cell and its localization strictly depends on FtsZ. Possibly, GroEL system plays a role in stabilizing the FtsZ for polymerization or in the recruitment of accessory proteins to the division site necessary for septa formation [35]. Apart from GroEL, GrpE, which also plays an important role in preventing aggregation of unfolded proteins [35], was found to be elevated after curcumin treatment along with the other stress proteins probably to stabilize the growth under stress condition.

In addition to the alteration of the cell division and cell wall synthesis, multiple vital anabolic and catabolic pathways in *B*. *subtilis* were found to be altered under curcumin treatment. Elevated expression levels of multiple components of the central carbon metabolism pathways, including most of the TCA cycle enzymes were observed, particularly during the late phases of drug exposure probably to provide the sufficient energy precursors for survival of the microorganism under adverse conditions (Fig 6). Consequently, PP pathway and other carbohydrate metabolism were also induced to provide the sufficient precursors for nucleotide biosynthesis (Fig 6). Interestingly, the expression level of S-adenosyl methionine synthase involved in synthesis of S-adenosyl methionine (SAM), which is one of the crucial co-factor involved in multiple vital cellular processes, was found to be reduced. In *E*. *coli*, it has been reported that SAM plays a vital role in methylating the late cell division proteins for recruitment to division site and deletion of the S-adenosyl methionine synthase causes elongation of the cells [36,37]. The reduced levels of SAM synthase suggest a similar effect on the late cell division proteins which may induce the elongation process. Additionally, cell membrane plays a crucial role in maintaining the membrane potential and PMF to generate energy. Fatty acid synthesis is essential for phospholipids and lipid bilayer synthesis, which is in-tern linked to the cell membrane permeability (Fig 6). Curcumin treatment significantly affected the expression of fatty acid synthesizing enzymes in correlation to the time of exposure, which indicates that reduced synthesis of fatty acids/phospholipid is required for the cell membrane [38,39] (Fig 4). Moreover, the functional assays clearly indicated the alteration of membrane permeability and lower respiratory activity under curcumin treatment (Fig 5A–5D). Recent studies also highlighted that membrane potential or PMF (proton motive force) is essential for the localization of cell division proteins towards the division site [40–42]. Additionally, membrane damage or membrane stress marker proteins such as phage shock protein A homolog (YdjF) and NH(3)-dependent NAD(+) synthetase (NadE) were found to be induced under curcumin treatment. Interestingly, similar responses were reported earlier with cell membrane targeting lantibiotics [21] indicating there might be some level of similarities in the mode of action of these antimicrobials.

Quite a few bio-physical and global proteome analyses of eukaryotic systems have been reported earlier to elucidate the mechanism of action of curcumin [43–46]. Even though the inhibitory concentration of curcumin required for bacteria and tumor cell lines are almost similar, curcumin is still considered as a potent antimicrobial compound due to two important reasons: firstly, curcumin is a very safe compound and human body can take up to 12g of curcumin/day and secondly, the poor bioavailability of curcumin in humans under physiological conditions [47, 48]. Therefore, the inhibitory concentration of curcumin required for restraining the microorganisms *in vivo* cannot adversely affect the host system.

In summary, this is the first comprehensive proteomic study indicating the modulation of cell wall synthesis, cell division, chaperones and central metabolism in *B*. *subtilis* due to curcumin treatment. Our findings indicate that inhibition of the cell division machinery is one of the prime targets of this drug; since, multiple proteins involved in cell division process such as FtsH, FtsL, MinJ, GpsB and DivIB were found to be differentially expressed as a consequence of the curcumin treatment. In addition, cell membrane permeability/potential has been affected drastically due to repression of fatty acid synthesizing enzymes which is further supported by reduced respiratory activity and leakage of ions. Quite a few earlier studies have highlighted that the membrane permeability or potential is essential for cell division by supporting the division proteins at the septum site. To this end, a detailed investigation of the functional properties of these differentially expressed proteins in the context of cell division will be useful to enhance our understanding regarding the mechanism of action of this potential antibacterial agent.

## Materials and Methods

### Growth Curve Analysis of *B*. *subtilis* under Curcumin Treatment

The present study was carried out with *B*. *subtilis* AH75 strain having a spectinomycin antibiotic marker [49]. Cultures were grown overnight at 37^0^ C in LB medium containing 100 μg/mL spectinomycin. The overnight culture was re-inoculated in fresh media with a final OD of 0.05 (at 600 nm) to adjust the cell population at 10^6^/mL. Growth of *B*. *subtilis* cultures were continued at 37° C in the absence and presence of IC_50_ (20μM) and MIC (100μM) concentrations of curcumin. The growth of the *B*. *subtilis* cultures was monitored by measuring the OD at 600 nm of an aliquot after every 20 min interval and continued up to 360 min (mid-exponential phase). This experiment was carried out in triplicates and the growth curve was plotted with mean values and standard deviation.

### Culture Conditions and Microscopic Analysis

Overnight cultures were sub-cultured into fresh LB media with sufficient amount of inoculums to get the final OD_600_ at 0.1 and further incubated again at 37^°^C till the OD_600_ reached to 0.2. Curcumin stock solution 500 mM (Sigma, St. Louis, MO, USA) was prepared in DMSO. Curcumin (IC_50_- 20 μM) was added to the bacterial cultures having 0.2 OD_600_ and subsequently incubated at 37^°^C. Control cultures were grown under the identical condition i.e. in the presence of the solvent (DMSO), but without the curcumin. Curcumin treated samples (10 mL each) were harvested at 20, 60 and 120 min of the drug treatment by centrifuging at 7000 rpm for 10 min at 4^°^C. The cells were fixed with 2.8% formaldehyde and 0.04% glutaraldehyde at 37^°^C for 30 min after washing with PBS buffer for 3 times. DAPI staining was performed by adding 1μg/μL concentration to the fixed cells for 20 min in dark. Morphological changes were observed under a fluorescence microscope (Axio observer Z1 microscope using Axiovision software, Zeiss) using 100 X objective.

### Sample Preparation from Whole Cell Extract

Protein extraction was performed from control and curcumin (20, 60 and 120 min treatment) treated *B*. *subtilis* AH75 strains using TRIzol extraction protocol [50]. Briefly, the bacteria were harvested at different time points of curcumin exposure (20, 60 and 120 min) and untreated control samples and washed with PBS buffer (pH 7.4) for 4 times to remove the media components. Cell lysis was performed with lysozyme (1 mg/mL) and sonication in presence of protease inhibitor cocktail (GE Healthcare). To the cell lysates, TRIzol and chloroform were added to remove RNA, and ethanol was added to remove DNA and chilled acetone was added to precipitate protein. Protein pellet was washed with guanidine-HCl and acetone to remove the phenol and salts. Protein pellets were air dried and finally dissolved in rehydration buffer containing 7 M urea, 2 M thiourea, 2% CHAPS (w/v) and traces of bromophenol blue. The protein concentration in each sample was measured using 2-D quant kit (GE healthcare) following the manufacturer’s instructions.

### Cy Dye Labelling and 2D-DIGE

Control and all three time points (20, 60 and 120 min) of curcumin treated samples were subjected to quantitative 2D-DIGE analysis. Prior to labelling, pH of the protein samples were adjusted to 8.5 with 100 mM NaOH. 60μg of each protein sample (control and all three time points of curcumin treated and internal standard) were individually labelled with 400 pmol of CyDyes (GE Healthcare). Subsequent to addition of CyDyes, samples were incubated on ice for 1 hr in the dark. Labelling reaction was stopped by addition of 10 mM lysine followed by additional 10 min incubation on ice. Dye-swapping was carried out while labelling the test and control samples to get rid of dye selection biasness. The details of labelling strategy are provided in S4 Table. Samples labelled with Cy3, Cy5 and Cy2 were mixed, diluted with the rehydration buffer having 1% DTT and 1% IPG buffer and loaded on 24 cm, 4–7 pH IPG strips and rehydration was performed for 14 hrs at room temperature. Isoelectric focussing and SDS-PAGE was performed with the settings described previously by Reddy et al., [50].

### Image Acquisition and DeCyder Analysis

Image acquisition and data analysis of 2D-DIGE gels were performed as described previously [51]. In brief, the 2D-DIGE gels were scanned at a 100 μm resolution using a Typhoon FLA 9500 biomolecular imager (GE healthcare) using suitable excitation/emission wavelengths for each of the CyDye (Cy3 (523/580nm), Cy5 (633/670nm) and Cy2 (488/520 nm). Comparative analysis and relative protein quantification between the curcumin treated and control samples (control vs 20 min, control *vs*. 60 min and control *vs*. 120 min curcumin treatment) was performed using DeCyder 2D software, version 7.0 (GE Healthcare). Two different modules, differential in-gel analysis (DIA) and biological variation analysis (BVA), of the DeCyder software were used for 2D-DIGE analysis. DIA module was used for spot detection and pair-wise comparisons of normal and curcumin treated samples to the mixed internal standard present in each gel. Further analysis was performed using BVA module to get the variation in protein expression between the two experimental groups (control and curcumin treated: 20/ 60/ 120 min). The differentially expressed and statistically significant (*p* ≤ 0.05) protein spots present in all the gels were excised and used for MS analysis.

### In-gel Digestion and Protein Identification using MALDI-TOF/TOF MS

In-gel digestion of the differentially expressed protein spots (*p* ≤ 0.05) was performed following the same protocol as mentioned by Shevchenko et al. and Reddy et al. with minor modifications [52, 50]. The extracted trypsin digested peptides were further processed using Zip-Tip C18 pipette tips (Millipore, USA) following the manufacturer’s protocol for enrichment of the peptides and removal of salts. The protein identification was performed with MALDI-TOF/TOF mass spectrometer (AB Sciex, Framingham, MA) linked to a 4000 series explorer software (v.3.5.3) as described previously [50]. data analysis was performed by using MASCOT version 2.1 (http://www.martixscience.com) search engine with following parameters were specified; database- SwissProt, *B*. *subtilis* taxonomy, trypsin digestion with single missed cleavage, oxidation of methionine as a variable modification and carbamidomethylation of cysteine residue as a fixed modification, mass tolerance 75 ppm for MS and 0.4 Da for MS/MS.

### In-solution Digestion, iTRAQ labelling and Peptide OFFGEL Fractionation

Protein samples extracted from biological triplicates of control and curcumin treated (all the three time points) *B*. *subtilis* cultures, used for DIGE analysis, were analyzed further using iTRAQ-based quantitative proteomics. Protein in rehydration solution was exchanged to TEAB buffer using Amicon Ultra 0.5 mL centrifugal 3 kDa filters (Millipore, Watford, UK). After buffer exchange, biological triplicate samples (control, 20 min, 60 min and 120 min pooled samples were pooled and quantified using QuickStart Bradford reagent (BioRad, USA). Prior to the iTRAQ labelling, in-solution digestion was performed (100 μg proteins from each sample) following the manufacturer’s instructions. The protein samples were reconstituted in dissolution buffer followed by reduction with (tris (2-carboxyethyl) phosphine (TCEP)) at 60°C for 1 h and subsequently alkylated using methyl methanethiosulfonate (MMTS) for 20 min at room temperature. Trypsin (Trypsin Gold, mass spectrometry grade, Promega, Madison, WI, USA) was added at a 1:20 trypsin: protein ratio and samples were incubated at 37° C for 16 hrs for digestion.

After in-solution digestion, iTRAQ (AB Sciex UK Limited, UK) labelling of the peptides was performed as per the manufacturer's instructions. The labeling strategy is as follows i.e. control-114, 20 min-115, 60 min-116 and 120 min-117 and incubated for 60 min at RT. Labelling was quenched using 100 μL of milliQ water and incubated at RT for 30 min. Peptide OFFGEL fractionation was performed using 3100 OFFGEL fractionator (Agilent Technologies, Santa Clara, CA) with high resolution (pH 3–10, 24 cm) IPG strip following manufacturer’s instruction for peptide fractionation. First, IPG strip was rehydrated for 30 min with 40 μL of rehydration buffer (water and pH 3–10 IPG buffer) in each well followed by adding 150 μL of sample to each well and focusing was performed for a total of 50 kV with maximum voltage of 4000 V and 50 μA current. Each fraction was collected separately and processed using C_18_ STAGE tips for removal of salts and other impurities prior to the MS/MS analysis.

### Q-TOF and LTQ-Orbitrap Analysis

ITRAQ-labelled samples were analyzed using two mass spectrometry platforms; Agilent 6550 QTOF and Thermo Scientific LTQ Orbitrap Velos. Quantitative proteome analysis was performed using 6550 ESI Q-TOF iFunnel technology (Agilent technology, USA) coupled with 1260 Infinity HPLC-nano-chip cube controlled by MassHunter Acquisition software. The nano chip contains analytical column (75 μm x 46 mm with 5 μm pore size) made of ZORBAX 300SB C18 with CII Filter followed by an enrichment column. Peptide sample from each fraction was analyzed by loading 5 μL of sample with the flow rate of 2 μL/min of capillary pump and 200 nL/min of nano pump. Peptide separation was performed using a gradient of 27 min; 5% sol-B at 0 min, 12% sol-B at 2 min; 30% sol-B at 20 min; 60% sol-B at 22 min, 95% sol-B at 24 min and 5% sol-B till 27 min. The acquisition parameters of mass spectrometry analysis were as follows: positive ion mode, MS mode: m/z range from 100–3200 with MS scan rate 2 spectra/sec, MS/MS mode: scan rate 5 spectra/sec, max precursor selection is 15 with charge state of >2 with collision energy maintained with slope-3.9 and offset-2.9.

LTQ-Orbitrap Velos mass spectrometer (Thermo Fischer Scientific, Bermen, Germany) interfaced with Proxeon Easy nLC system (Thermo Scientific, Bremen, Germany) was used for analysis of the iTRAQ-labelled OFFGEL fractionated samples. Peptides were enriched on a trap column (75μm x 2cm) packed in-house using C18 material (Magic C18AQ, 5um, 100A, Michrom Biosciences Inc.) with a flow rate of 3μL/min and resolved on an analytical column (75 μm x 10 cm, Magic C18AQ, 3um, 100A, Michrom Biosciences Inc.) at a flow rate of 350 nL/min using a linear gradient of 7–30% acetonitrile over 60 min. Precursor MS scan (from m/z 350–1,800) and MS/MS was acquired with a mass resolution of 60,000 and 15,000 at 400 m/z in orbitrap mass analyzer. In each duty cycle twenty most intense peaks were selected for MS/MS fragmentation using higher-energy collision dissociation (HCD) mode at 41% normalized collision energy and isolation width was set to 1.9 m/z. Singly charged and unassigned charge precursor were rejected. Dynamic exclusion settings were enabled and acquired ions were excluded for 45 sec. The automatic gain control for full MS and MS/MS was set to 1 × 10^6^ and 5 × 10^4^ ions, respectively. The maximum ion accumulation time was set to 200 msec for MS and 300 msec for MS/MS scans. The lock mass option was enabled using polysiloxane ion (m/z, 445.120025) from ambient air for internal calibration.

### Protein Identification, Quantitation and Data Availability

Raw data obtained from both Q-TOF and LTQ-Orbitrap mass spectrometer processed using Proteome Discoverer 1.3 (Thermo Fischer Scientific, Bermen, Germany). The data was searched using SEQUEST algorithm against UniProt *B*. *subtilis* 168 reference protein database having 4227 reviewed protein sequences. While performing the database search, following parameters were specified: 20 ppm precursor mass tolerance, 0.1Da fragment mass error tolerance, trypsin as proteolytic enzyme permissible with one missed cleavage and iTRAQ modification at N-terminal of peptide and lysine as fixed and oxidation of methionine as variable modification, respectively. High peptide confidence and top peptide rank filters were used to extract the peptide and protein data. The false discovery rate (FDR) was calculated based on decoy database search and a cut-off of 1% was used to report identifications. Protein data was normalized with “normalize on protein median” with minimum protein count as 20 proteins. LTQ-orbitrap mass spectrometry proteomics data have been deposited at the ProteomeXchange Consortium [53] via the PRIDE partner repository with the dataset identifier PXD000644.

### Functional Pathway Analysis

The differentially expressed *B*. *subtilis* proteins identified in both quantitative iTRAQ analysis with similar trend (1% FDR, fold change- 1.5) were further subjected to *in silico* analysis using DAVID version 6.7 (Database for Annotation, Visualization and Integrated Discovery; http://david.abcc.ncifcrf.gov/home.jsp) and KOBAS 2.0 (KEGG Orthology Based Annotation System; http://kobas.cbi.pku.edu.cn/home.do) [54, 55]. The pathway analysis of DAVID module was used to build the pathways associated with the differentially expressed proteins indentified in *B*. *subtilis* after curcumin treatment. In addition, pathways were built through KOBAS *v* 2.0 using the UniProt accession IDs of the differentially expressed candidates identified from all three different time points (fold change -1.5 and 1% FDR) [56].

### Resazurin Microtiter Assay for Metabolic Activity

Resazurin assay was performed as described by Mariscal et al., to check the cell viability and metabolic activity of *B*. *subtilis* after curcumin treatment [57]. Triplicates of control and IC_50_ (20 μM) curcumin treated *B*. *subtilis* cultures at 20 min, 60 min and 120 min were harvested. Optical density of the culture was measured for determining the cell population. Cultures were diluted with PBS buffer and four different dilutions having cell population from 10^6^ to 10^8^ cells/ mL were used for the assay. Resazurin stock was added to the each sample and the fluorescence intensity of resorufin was monitored at 590 nm for every 15 sec for next 30 min in a real-time PCR machine (MyiQ2 system, BioRad, USA). Mean values of curcumin treated and control groups were compared by using Student's t-test test and a *p*-value < 0.05 was considered as statistically significant.

### Respiratory Activity Assay using CTC

Respiratory activity assay using 5-Cyano-2,3-di-(p-tolyl)tetrazolium chloride (CTC) was performed as described by Rodriguez et al.,[58]. 50 mM stock of CTC (5-cyano-2,3-ditolyl tetrazolium chloride) was prepared in ultrapure water and the final concentration of 5 mM was used for the assay. Control and curcumin treated (20 min, 60 min and 120 min) cultures were used for the assay after washing the pellets with PBS buffer. CTC stock (100 μL) was added to 900 μL of the cultures dispersed in PBS buffer and incubated at 37^°^C for 30 min in dark condition with mild agitation. Then, the cultures were fixed with 2.8% formaldehyde and 0.04% glutaraldehyde, and counter stained with DPAI (1 μg/mL) and incubated on ice for 20 min. Negative control was prepared by disrupting the membrane with 2.8% formaldehyde and 0.04% glutaraldehyde for 30 min prior to the addition of CTC and subsequently counter stained with DAPI (1 μg/mL). Stained *B*. *subtilis* cultures (curcumin treated and untreated, and negative control) were subjected to FACS (fluorescence-activated cell sorting) analysis (FACS Aria; Becton Dickinson, San Jose, CA, USA). The instrument was operated on logarithmic scale by keeping rest of the parameters fixed. Blue (358 nm) and red fluorescence (630 nm) lasers were used for DAPI and CTC, respectively. FACS data analysis was performed using Cyflogic flow cytometry data analysis software, version 1.2.1 (http://www.cyflogic.com/) after measuring total 20,000 events.

### Measurement of Potassium and Phosphate Release after Drug Treatment

Potassium (K^+^) and inorganic phosphate (P) leakage measurement was performed using Inductively Coupled Plasma—Atomic Emission Spectrometer (ICP-AES). *B*. *subtilis* cultures were grown till mid-exponential phase. The cells were harvested by centrifugation and the pellet was washed four times with Na-HEPES buffer (pH 7.0) and then resuspended in same buffer. Bacterial cultures were inoculated into Na-HEPES buffer having 10 mM glucose with a final OD_600_ of 1.0. Curcumin (IC_50_ and 2X IC_50_) treated and untreated culture having only DMSO (as a control) were analyzed by K^+^ and P leakage assay. Samples were collected at 20 min, 60 min, 90 min and 120 min time intervals from both IC_50_ and 2 x IC_50_ treated samples after the addition of curcumin. Positive control was prepared by heating the control samples for 30 min at 70°C [59]. The cells were pelleted down by centrifugation at 10,000 x g for 10 min and the supernatant was collected for measuring the extracellular potassium and phosphate levels using ICP-AES. The standard curve was prepared for both K^+^ and P and used it for measuring unknown concentrations.

### Quantitative RT-PCR for Expression Analysis

*B*. *subtilis* AH75 cultures treated with IC_50_ curcumin at 20, 60 and 120 min and untreated control cultures (20 mL culture each) were used for total RNA extraction using TRIzol reagent (Invitrogen, USA). Quality of the extracted RNA was evaluated on denatured 1% agarose gel and by measuring the spectrometric 260/280 ratio in a Nanodrop (Implen, Germany). cDNA synthesis was performed with 4 μg of total RNA by using RevertAid first strand kit (Fermentas, Europe) following the manufacturer’s protocol. Prior to real-time expression analysis, quality of cDNA synthesis was checked with PCR using designed primers (Table 3). The real-time expression analysis for selected genes (*MurAA*, *FtsH and SpoVG*) was performed using Eco real-time PCR system (Eco real Time PCR, Illumina). Following setting was used for RT-PCR: denaturing step at 95°C for 5 min and 40 cycles of 95°C for 30 sec, annealing at 46°C for 30 sec and extension at 72°C for 30 sec followed by melting curve for 15 sec at 95°C, 15 sec at 46°C and finally 95°C for 15 sec. Analysis of expression levels of the genes was carried as a relative quantification by taking 16S rRNA as an internal control to normalize the data among the different triplicates.

**Table 3:** List of the primers used for quantitative RT-PCR analysis for selected genes.

Gene	Forward primer (5'-3')	Reverse primer (5'-3')	Annealing temperature (°C)	Amplicon size (bp)
MurAA	5'TACAGGTCATGCAAGAGT-3'	5'TTCTCTGTAGCTCCTACACT-3'	46	196 bp
FtsH	5'CACCGTTATCGGTCTCGTTT-3'	5'CCAAGAGGCCGACAATTTTA-3'	46	158 bp
SpoVG	5'TTCGTGTGATTGATGGAAACA-3'	5'TGCTTCAGTGTCACCCAGAC -3'	46	164 bp
16S r RNA	5'GATCTTAGTTGCCAGCATTC-3'	5'TTACTAGCGATTCCAGCTTC-3'	46	233 bp

### Biosensor-based Interaction Analysis

BIAcore T200 system (GE healthcare) was used to study the interaction between FtsZ and curcumin. The purified FtsZ was immobilized on a CM5 sensor chip using amino coupling chemistry (5000 RU). Eight different concentrations of curcumin (1.25, 2.5, 5, 10, 20, 40, 80 and 160 μM and 40 μM used in duplicate) were injected with 1.05X HBS-EP^+^ in 5% DMSO buffer (10 mM HEPES, 150 mM NaCl, 3 mM EDTA, 0.005% surfactant P40, pH 7.4) with flow rate of 30 μl/min, contact time 120 sec and dissociation time 300 sec. The interactions were monitored in a real-time manner by measuring the response unit (RU). A mock surface was also used to see the binding of curcumin to the matrix (CM-5 sensor chip surface) and the analyzed results were subtracted from the blank surface. In addition, we have made solvent correction curve with DMSO (4.5% and 5.8% DMSO) to normalize the results (curcumin was dissolved in 5% DMSO).

## Supporting Information

S1 Fig (A) Growth curve analysis of B. subtilis AH75 in the presence and absence of DMSO. (B) I, II, III are the DIA, DAPI and overlay images in the B. subtilis in the absence of DMSO and IV, V and VI are the DIA, DAPI and overlay images in the B. subtilis in the presence of DMSO
(TIF)

S2 Fig Differentially expressed proteins identified in DIGE and their 3D and BVA graph views. **(A)** Differential expression of proteins at 60 min and **(B)** Differential expression of proteins at 120 min.
(TIF)

S3 Fig S-curve analysis for differential expression protein comparison among technical triplicate runs (Q-TOF data). (TIF)

S4 Fig (A) Venn diagram showing the unique and overlapping differentially (both up and down regulated proteins separately) expressed proteins identified by LTQ-Orbitrap and QTOF instruments. **(B)** Venn diagrams showing the unique and common differentially expressed proteins (total identified proteins) identified in LTQ-Orbitrap and Q-TOF data.
(TIF)

S5 Fig (A) Venn diagram showing the unique and overlapping differential expressed proteins among LTQ-Orbitrap, Q-TOF and 2D-DIGE data. (B) Comparison of data between LTQ-Orbitrap and 2D-DIGE (all the identified proteins). In case of 60 min treatment, diaminopimelate epimerase was identified only in DIGE but not in iTRAQ analysis.
(TIF)

S6 Fig Venn diagram showing the unique and overlapping proteins among the time point analysis in both LTQ-Orbitrap and Q-TOF data. (TIF)

S7 Fig Quantitative profiles of the differentially expressed proteins (identified in iTRAQ-based quantitative proteomics analysis using Q-TOF) involved in diverse biological processes. (TIF)

S1 Table MADLI-TOF/TOF identification of the proteins from the differentially expressed spots identified in 2D-DIGE and their sequences information. (DOCX)

S2 Table Complete peptide and protein identification and quantitative iTRAQ data obtained from LTQ-Orbitrap and Q-TOF and their comparison. (XLSX)

S3 Table Different biological pathways modulated due to curcumin treatment identified in KOBAS and DAVID analysis. (XLSX)

S4 Table Cy dye labelling strategy for curcumin treated and control *B*. *subtilis* cultures for differential proteomics analysis using 2D-DIGE. (DOCX)
