# Correction: Comprehensive Analysis of Temporal Alterations in Cellular Proteome of *Bacillus subtilis* under Curcumin Treatment

**DOI:** 10.1371/journal.pone.0130782

**Published:** 2015-06-17

**Authors:** Panga Jaipal Reddy, Sneha Sinha, Sandipan Ray, Gajanan J. Sathe, Aditi Chatterjee, T. S. Keshava Prasad, Snigdha Dhali, Rapole Srikanth, Dulal Panda, Sanjeeva Srivastava

S7 Fig is incorrect. Please view the correct S7 Fig below.

There are errors in Table 2. The values provided for Q-TOF data were inadvertently changed. Please see the corrected Table 2 here.

**Table 2:** Partial list of differentially expressed proteins in *B*. *subtilis* due to curcumin treatment obtained from iTRAQ analysis*

UniProt ID	Name of the protein	Gene name	Coverage	Uni. peptides(Orbitrap)	Fold change (Orbitrap)			Uni. peptides(Q-TOF)	Fold change (QTOF)		
					20 min	60 min	120 min		20 min	60 min	120 min
**Cell wall synthesis**											
P70965	UDP-N-acetylglucosamine 1-carboxyvinyltransferase 1	MurAA	35.32	13	0.89	0.64	0.41	13	0.886	0.609	0.303
Q03522	UDP-N-acetylmuramoylalanine—D-glutamate ligase	MurD	31.04	13	0.98	0.67	0.47	4	1.145	0.979	0.824
P40778	UDP-N-acetylmuramate—L-alanine ligase	MurC	25.46	9	1.03	0.71	0.54	6	1.173	1.528	0.522
Q03523	UDP-N-acetylmuramoyl-L-alanyl-D-glutamate—2,6-diaminopimelate ligase	MurE	37.04	14	1.02	0.74	0.55	9	0.839	0.807	0.301
P37585	UDP-N-acetylglucosamine—N-acetylmuramyl-(pentapeptide) pyrophosphoryl-undecaprenol N-acetylglucosamine transferase	MurG	37.47	11	0.91	0.8	0.63	3	0.671	0.678	0.402
P94556	Glutamate racemase 1	RacE	25.37	4	0.97	0.84	0.68	3	0.974	1.024	0.487
P96613	UDP-N-acetylmuramoyl-tripeptide—D-alanyl-D-alanine ligase	MurF	30.20	8	1.10	0.79	0.87	3	1.689	0.646	0.752
P0CI73	Glutamine—fructose-6-phosphate aminotransferase [isomerizing]	GlmS	30.33	15	0.92	0.79	0.53	10	0.931	0.751	0.396
P14192	Bifunctional protein GlmU	GlmU	23.03	8	0.93	0.83	0.58	7	1.397	0.916	0.819
**Cell division and Sporulation**											
P45693	Stage V sporulation protein S	SpoVS	38.37	2	1.10	2.59	6.10	3	5.896	5.730	7.008
P28015	Putative septation protein SpoVG	SpoVG	42.27	4	0.96	1.51	4.49	2	0.955	0.713	2.268
Q07867	Cell division protein FtsL	FtsL	8.55	1	0.72	1.41	3.09	NI	NI	NI	NI
P0CI74	Cell cycle protein GpsB	GpsB	43.88	4	0.98	1.06	1.46	2	1.042	1.439	1.621
P06628	Sporulation initiation phosphotransferase F	SpoOF	23.39	2	0.79	1.75	2.93	2	0.570	1.782	2.644
Q01368	Stage III sporulation protein AB	SpoIIIAB	5.26	1	0.89	1.29	2.02	NI	NI	NI	NI
P06534	Stage 0 sporulation protein A	SpoOA	13.86	3	0.97	1.33	2.09	1			1.190
P71088	Sporulation-control protein spo0M	SpoOM	37.21	10	1.23	1.00	1.61	5	1.135	0.964	1.712
P39624	Spore coat polysaccharide biosynthesis protein SpsD	SpsD	4.84	1	0.97	0.43	1.13	NI	NI	NI	NI
P37470	Peptidyl-tRNA hydrolase	SpoVC	13.83	2	1.10	0.82	0.61	3	1.844	0.577	0.570
**Fatty acid synthesis**											
O34746	3-oxoacyl-[acyl-carrier-protein] synthase 3 protein 1	FabHA	45.83	13	1.01	0.56	0.38	6	1.048	0.578	0.414
O07600	3-oxoacyl-[acyl-carrier-protein] synthase 3 protein 2	FabHB	18.15	6	0.94	0.54	0.41	2	0.945	0.341	0.200
P71019	Malonyl CoA-acyl carrier protein transacylase	FabD	48.26	14	0.93	0.64	0.44	13	0.916	0.558	0.457
P54616	Enoyl-[acyl-carrier-protein] reductase [NADH] FabI	FabI	51.16	11	0.91	0.65	0.54	5	0.999	0.741	0.648
O34340	3-oxoacyl-[acyl-carrier-protein] synthase 2	FabF	48.67	14	1.03	0.73	0.57	11	0.927	0.747	0.732
P51831	3-oxoacyl-[acyl-carrier-protein] reductase FabG	FabG	61.79	12	1.01	0.71	0.58	7	0.985	0.801	0.677
**Stress response**											
P37571	Negative regulator of genetic competence ClpC/MecB	ClpC	59.88	45	1.20	1.65	2.43	28	1.102	1.546	1.998
P80244	ATP-dependent Clp protease proteolytic subunit	ClpP	43.15	8	1.09	2.07	2.26	4	1.168	1.639	2.352
P39778	ATP-dependent protease ATPase subunit ClpY	ClpY	35.55	14	1.17	1.41	1.82	5	1.552	1.520	2.026
O31673	ATP-dependent Clp protease ATP-binding subunit ClpE	ClpE	16.17	5	0.95	1.72	1.76	NI	NI	NI	NI
P39070	ATP-dependent protease subunit ClpQ	ClpQ	21.55	4	1.13	1.08	1.61	NI	NI	NI	NI
P54617	Phage shock protein A homolog	YdjF	58.59	11	0.76	1.22	3.09	6	1.062	1.549	2.386
P54375	Superoxide dismutase [Mn]	SodA	67.82	9	1.11	1.59	2.69	6	1.055	1.303	2.161
P42297	Universal stress protein YxiE	YxiE	45.27	5	1.19	0.95	2.34	3	0.907	0.880	1.397
P51777	Cold shock protein CspD	CspD	89.39	5	0.92	1.22	2.25	3	0.997	1.243	1.452
P28599	10 kDa chaperonin	GroS	73.40	7	1.09	1.82	2.20	5	1.148	1.651	2.493
P28598	60 kDa chaperonin	GroL	74.63	39	1.07	1.69	2.10	24	1.079	1.498	1.955
P39158	Cold shock protein CspC	CspC	59.09	5	0.22	0.82	1.86	1	0.181	0.832	1.756
P81100	Stress response protein SCP2	YceC	37.69	6	1.05	1.30	1.74	2	0.998	1.194	1.857
P54377	Probable glycine dehydrogenase [decarboxylating] subunit 2	GcvPB	9.43	3	1.20	2.15	1.67	1	0.698	2.985	2.562
O32221	Copper chaperone CopZ	CopZ	68.12	3	0.66	1.04	1.63	1		1.358	0.897
P15874	Protein GrpE	GrpE	57.75	10	0.96	1.13	1.51	4	1.710	1.716	1.163
P80875	General stress protein 16U	YceD	49.74	6	1.05	1.30	1.50	6	0.980	1.294	1.469
**TCA cycle**											
P39120	Citrate synthase 2	CitZ	31.18	11	1.20	3.99	7.65	7	1.448	3.869	6.546
P09339	Aconitate hydratase	CitB	33.22	25	1.14	3.94	4.56	12	1.449	4.064	3.887
P39126	Isocitrate dehydrogenase [NADP]	Icd	44.21	20	1.10	3.06	3.53	9	1.003	3.302	2.885
P23129	2-oxoglutarate dehydrogenase E1 component	OdhA	35.28	27	1.37	2.90	1.96	14	1.113	1.911	1.634
P16263	Dihydrolipoyllysine-residue succinyltransferase component of 2-oxoglutarate dehydrogenase complex	OdhB	54.44	18	1.18	2.51	3.30	13	1.101	1.669	2.818
P80865	Succinyl-CoA ligase [ADP-forming] subunit alpha	SucD	33.00	6	1.12	2.73	3.39	4	2.458	2.642	3.074
P80886	Succinyl-CoA ligase [ADP-forming] subunit beta	SucC	55.32	22	1.11	3.26	4.08	14	1.185	3.907	3.806
P08065	Succinate dehydrogenase flavoprotein subunit	SdhA	49.32	22	1.44	2.40	2.06	9	1.279	1.914	2.462
P08066	Succinate dehydrogenase iron-sulfur subunit	SdhB	27.27	6	1.33	2.14	1.71	3	1.470	2.157	1.502
**Nucleotide biosynthesis**											
P29726	Adenylosuccinate synthetase	PurA	26.05	10	1.12	0.76	0.55	6	1.569	0.736	1.302
P12047	Adenylosuccinate lyase	PurB	35.27	15	0.95	0.77	0.45	4	1.135	0.855	0.737
P12046	Phosphoribosylaminoimidazole-succinocarboxamide synthase	PurC	4.98	1	0.61	0.95	0.62	NI	NI	NI	NI
P12039	Phosphoribosylamine—glycine ligase	PurD	12.56	4	0.72	0.91	0.62	NI	NI	NI	NI
P12044	N5-carboxyaminoimidazole ribonucleotide mutase	PurE	19.75	2	0.81	1.02	0.57	2	0.762	0.747	1.097
P12048	Bifunctional purine biosynthesis protein PurH	PurH	27.54	11	0.66	0.98	0.56	5	0.597	1.050	0.384
P12042	Phosphoribosylformylglycinamidine synthase 2	PurL	3.10	2	0.55	0.82	0.53	NI	NI	NI	NI
P29727	GMP synthase [glutamine-hydrolyzing]	GuaA	58.67	27	1.03	0.64	0.42	16	1.074	0.652	0.493
O05269	GMP reductase	GuaC	48.16	10	1.06	1.00	0.49	4	1.495	0.572	0.608
P14193	Ribose-phosphate pyrophosphokinase	Prs	35.65	11	0.93	0.74	0.52	8	0.834	0.911	0.720

* This is a partial list having selected candidates; complete list is provided in supplementary table S2

# Only present in one replicates of iTRAQ data

NI- Not identified in Q-TOF

## Supporting Information

S7 Fig Quantitative profiles of the differentially expressed proteins (identified in iTRAQ-based quantitative proteomics analysis using Q-TOF) involved in diverse biological processes. (TIF)
