# Supplementary figures and images for: Comprehensive Analysis of Temporal Alterations in Cellular Proteome of Bacillus subtilis under Curcumin Treatment
Source: PLoS One. 2015 Apr 14;10(4):e0120620. doi: 10.1371/journal.pone.0120620 (PMC4397091; doi:10.1371/journal.pone.0120620)

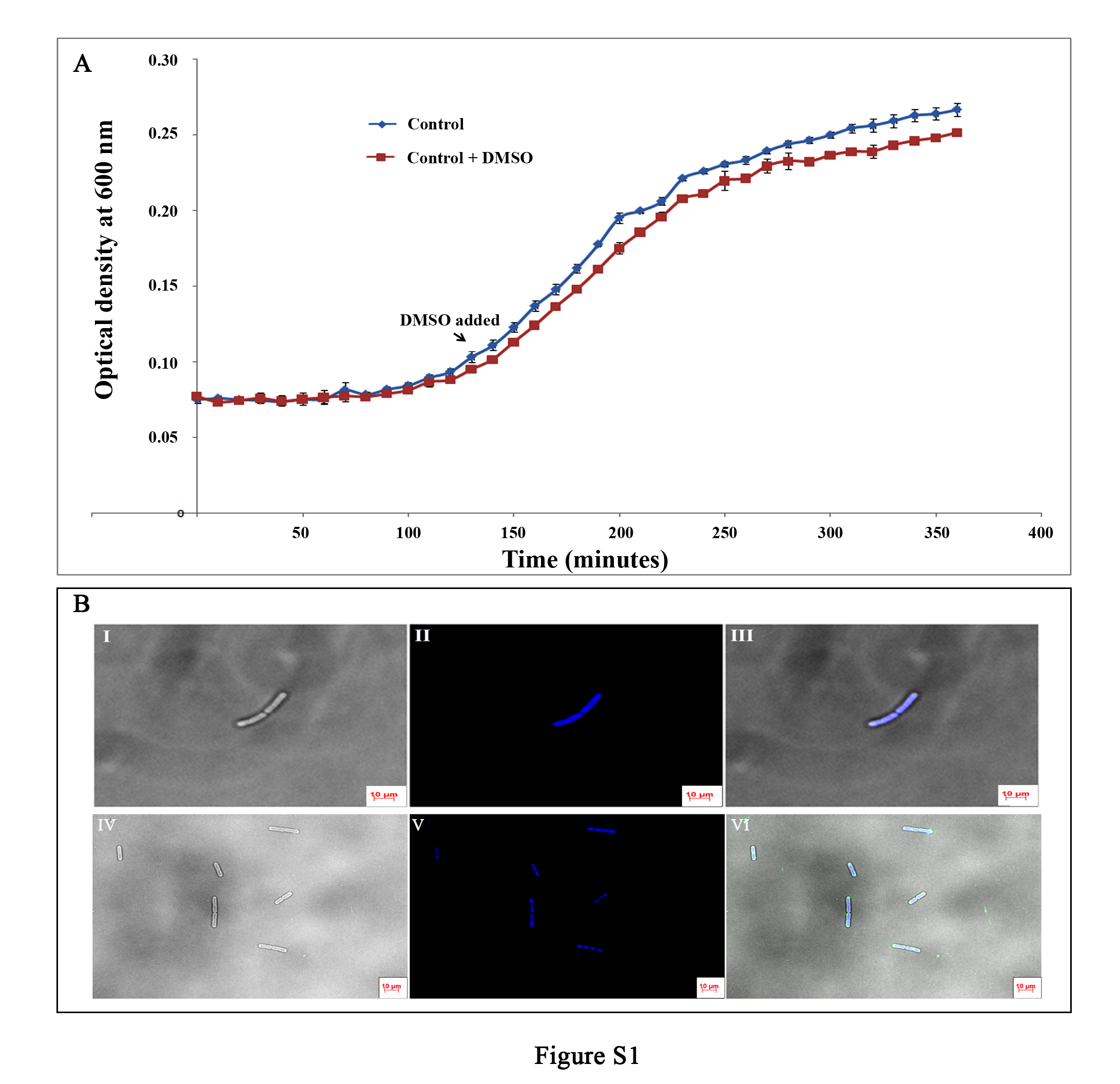

Supplement: S1 Fig — (B) I, II, III are the DIA, DAPI and overlay images in the B. subtilis in the absence of DMSO and IV, V and VI are the DIA, DAPI and overlay images in the B. subtilis in the presence of DMSO (TIF) [file pone.0120620.s001.tif]

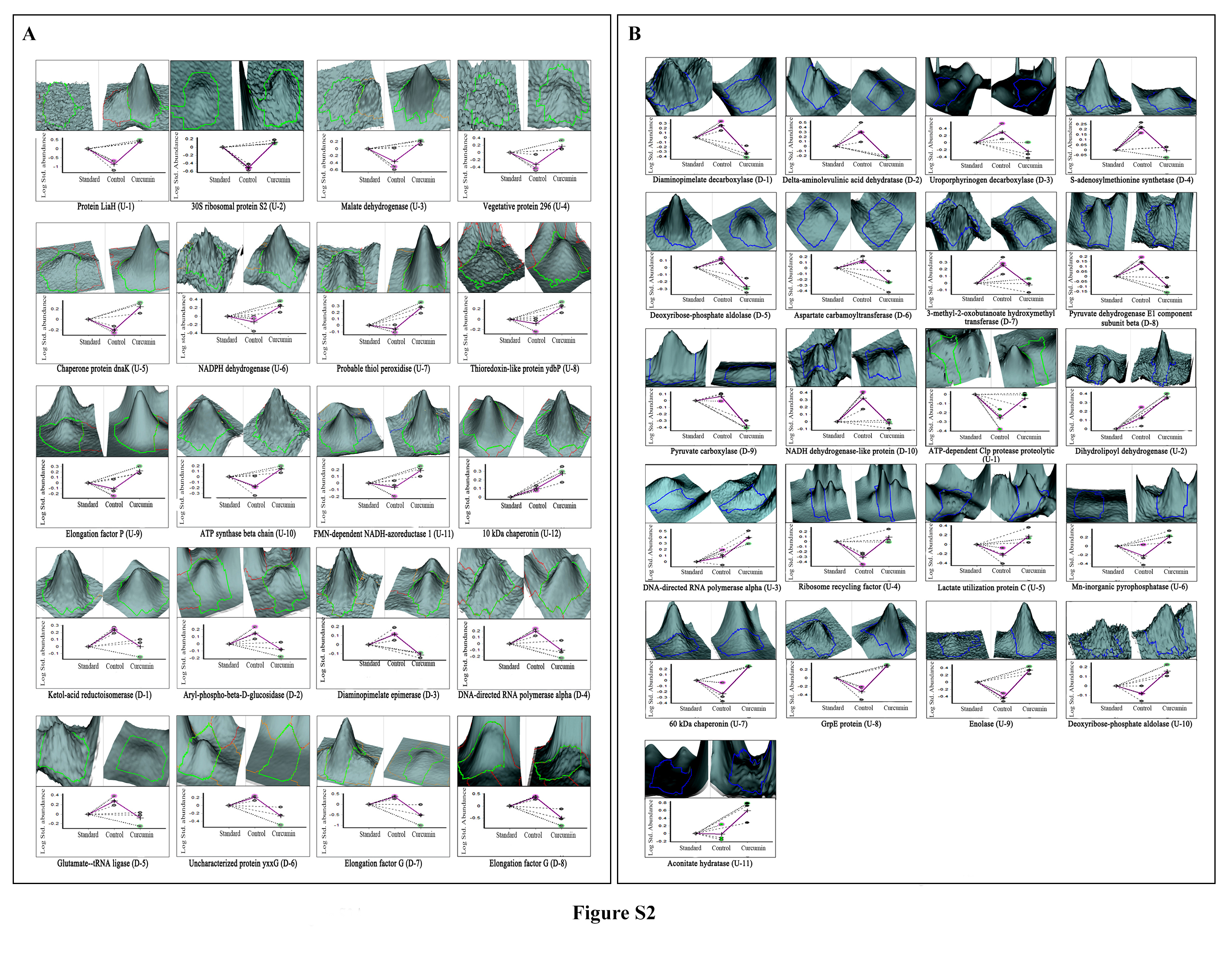

Supplement: S2 Fig — (A) Differential expression of proteins at 60 min and (B) Differential expression of proteins at 120 min. (TIF) [file pone.0120620.s002.tif]

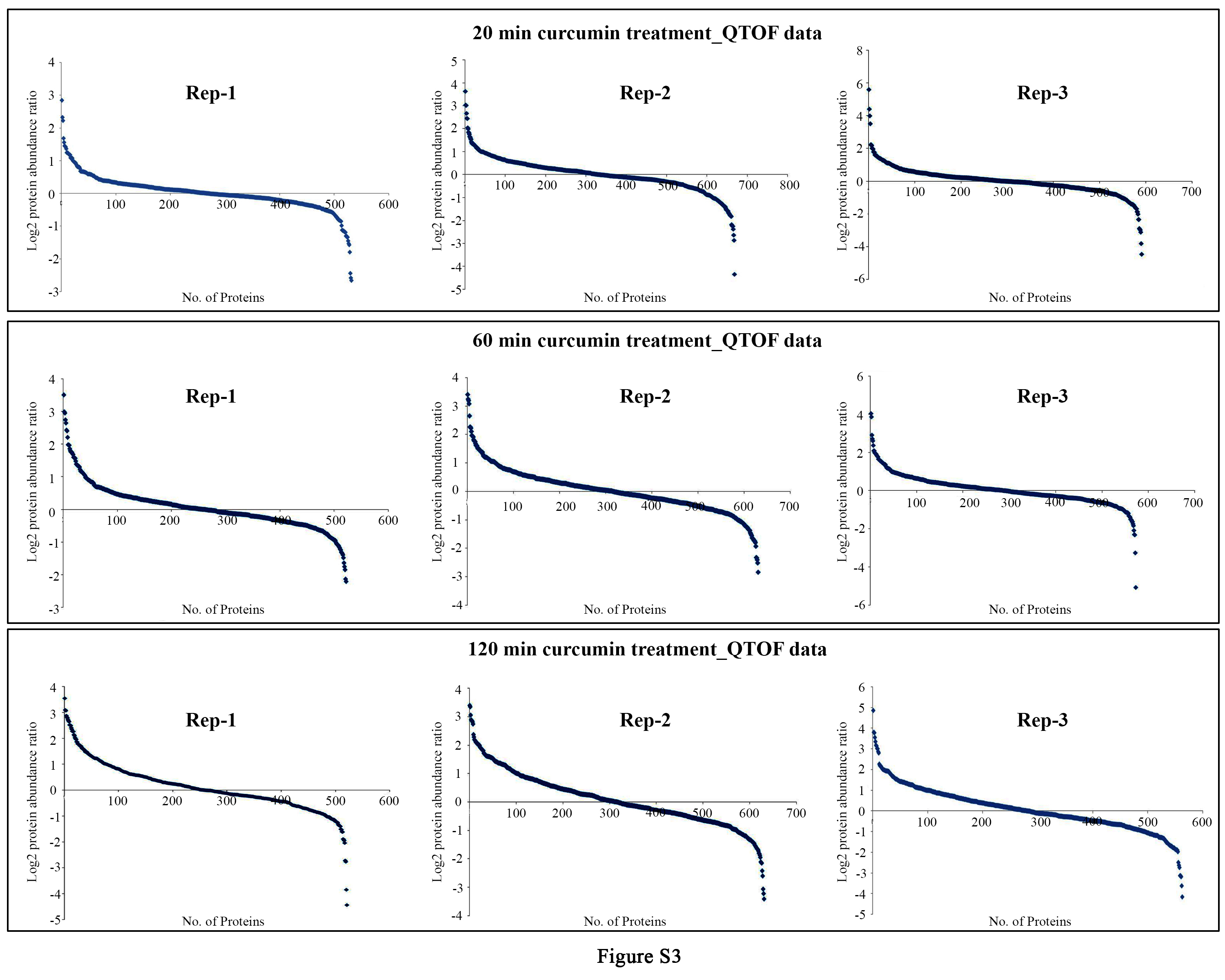

Supplement: S3 Fig — (TIF) [file pone.0120620.s003.tif]

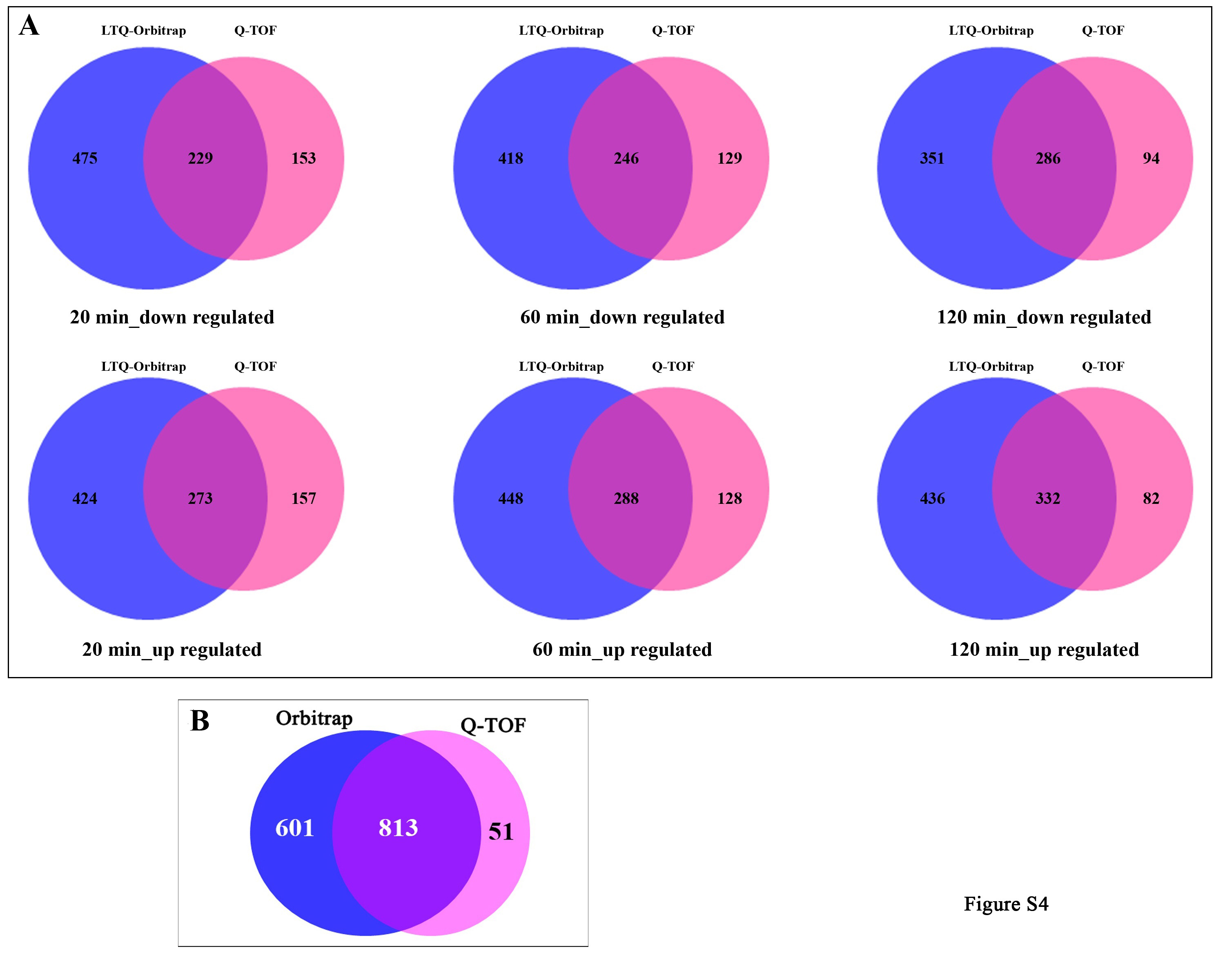

Supplement: S4 Fig — (B) Venn diagrams showing the unique and common differentially expressed proteins (total identified proteins) identified in LTQ-Orbitrap and Q-TOF data. (TIF) [file pone.0120620.s004.tif]

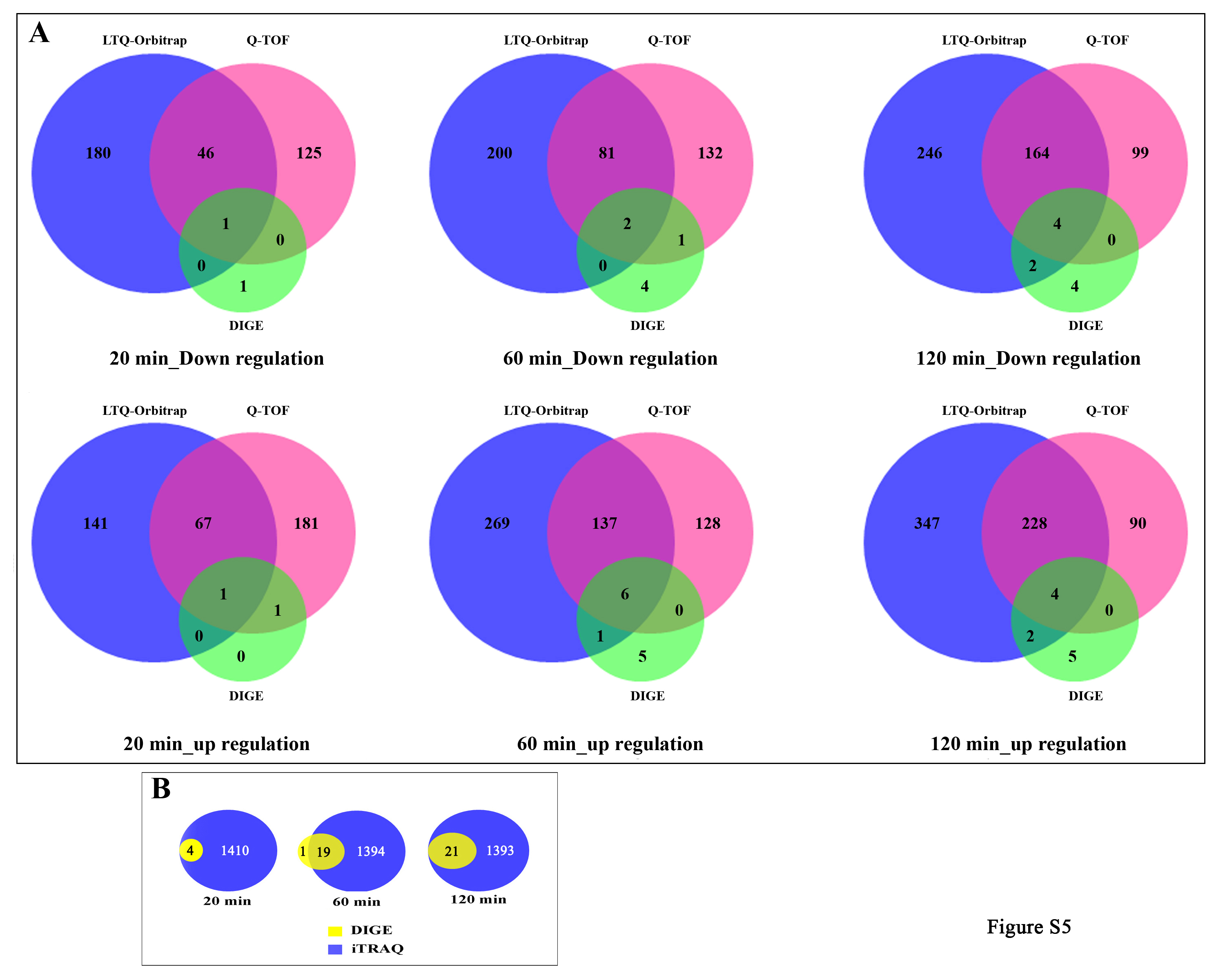

Supplement: S5 Fig — (B) Comparison of data between LTQ-Orbitrap and 2D-DIGE (all the identified proteins). In case of 60 min treatment, diaminopimelate epimerase was identified only in DIGE but not in iTRAQ analysis. (TIF) [file pone.0120620.s005.tif]

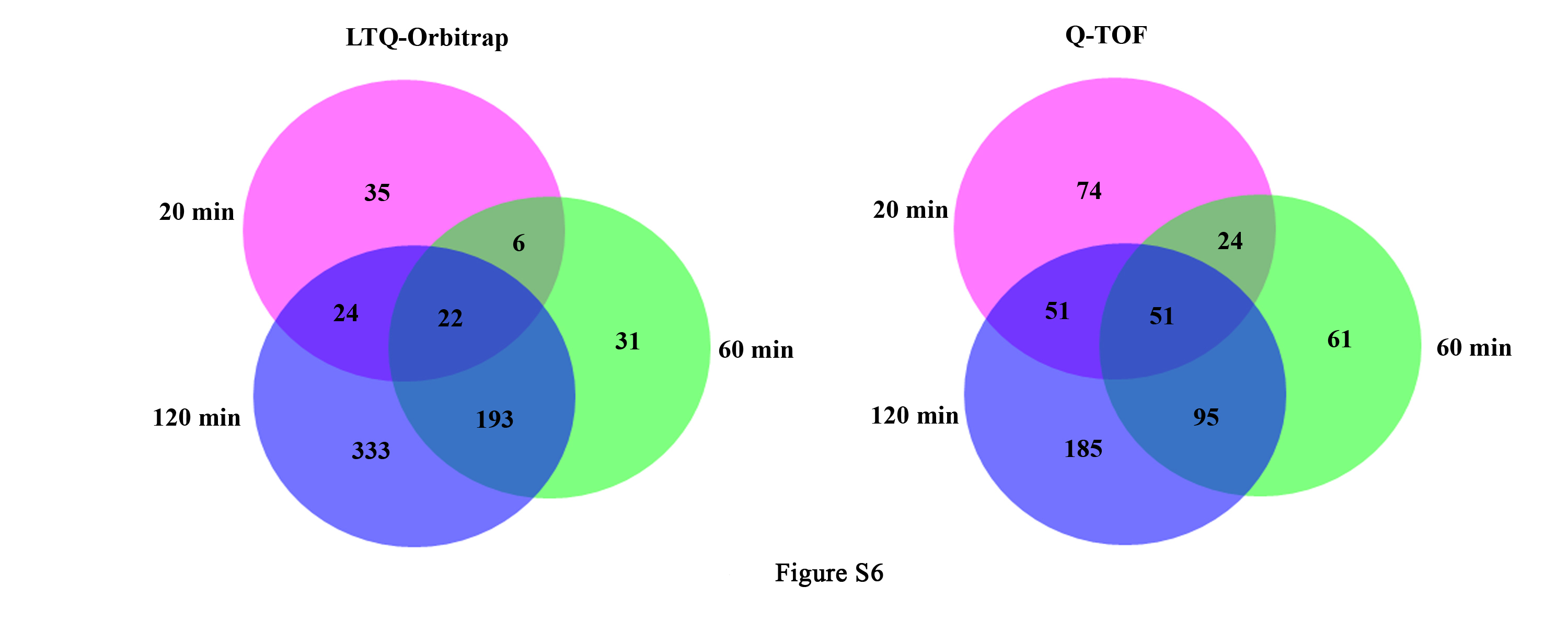

Supplement: S6 Fig — (TIF) [file pone.0120620.s006.tif]

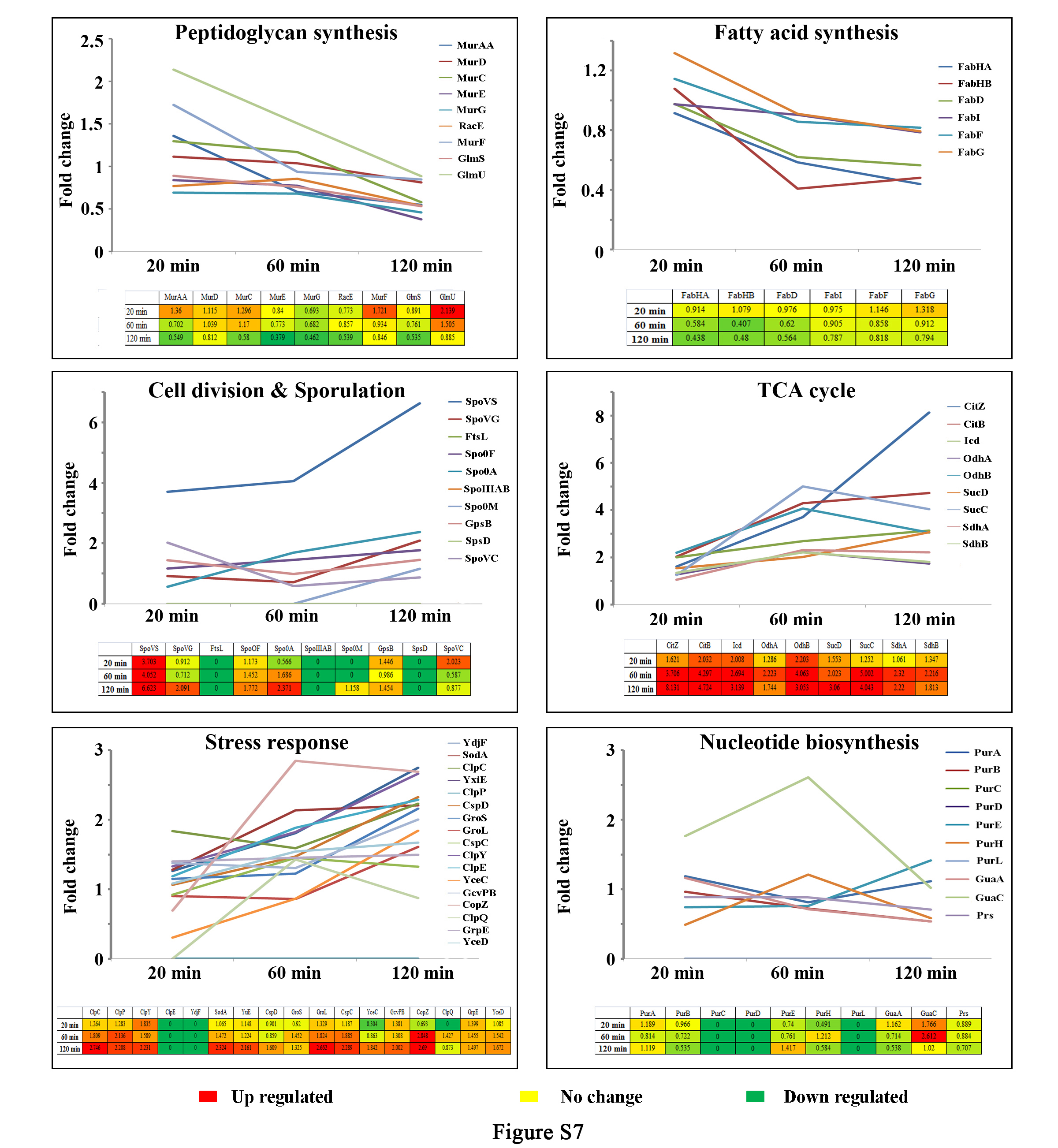

Supplement: S7 Fig — (TIF) [file pone.0120620.s007.tif]
