# Supplementary material for: Comprehensive Analysis of Temporal Alterations in Cellular Proteome of Bacillus subtilis under Curcumin Treatment
Source: PLoS One. 2015 Apr 14;10(4):e0120620. doi: 10.1371/journal.pone.0120620 (PMC4397091; doi:10.1371/journal.pone.0120620)
Supplement: S1 Table — (DOCX) [file pone.0120620.s008.docx]

**Table S1A: MALDI-TOF/TOF identification of differential expressed proteins from 20 min curcumin time point using DIGE analysis**

| **Spot No** | **Uniprot ID** | **Name of the protein** | **M.W (kDa)** | **No. of peptides** | **Fold change** | **Score** | **Ion Score** | **Peptides** |
| --- | --- | --- | --- | --- | --- | --- | --- | --- |
| **Down regulation** | | | | | | | | |
| D-1* | P37809 | ATP synthase subunit beta | 51.42 | 19 | -1.56 | 662 | 423 | EVQSTLQR  YDHLPEDAFR  VSQVLGPVVDVR  FTQAGSEVSALLGR  VALTGLTMAEHFR  TAMVFGQMNEPPGAR  FEDGHLPEIYNAIK  ALAPEIVGEEHYAVAR  DVQGQDVLFFIDNIFR  KLTEMGIYPAVDPLASTSR  GMEAVDTGAPISVPVGDVTLGR  VFNVLGENIDLNEPVPADAK  TREGNDLFYEMSDSGVINK  IQFFLSQNFHVAEQFTGQK  MPSAVGYQPTLATEMGQLQER  RIQFFLSQNFHVAEQFTGQK  ELQDIIAILGMDELGEEDKLVVHR  TVLIQELINNIAQEHGGISVFAGVGER  ISQPAASENEVGIDLTLEVALHLGDDTVR |
| **D-2** | P80698 | Trigger factor | 47.48 | 11 | -1.78 | 579 | 426 | AIDFLVENR  QEGNEGVLTVEVDAETFK  AVEEAGIEPVDRPEIDVEK  ASENAEIDVPQAMVDTELDR  FGVEALYQDALDILLPVEYPK  DVEVTFPEEYHAEDLAGKPAVFK  LQMQGMNLELYTQFSGQDEAALK  EEGAVEEGNTVVLDFEGFVDGEAFEGGK  ELPELDDEFAKDIDEEVETLAELTEK  AENYSLEVGSGSFIPGFEDQLVGLEAGAEK  LQMQGMNLELYTQFSGQDEAALKEQMK |
| **Up regulation** | | | | | | | | |
| U-1* | P46336 | Protein IolS | 35.17 | 13 | 1.85 | 167 | 104 | TFFPYTK  KSVDESLK  SEELIGEVLR  RADQLIDNIK  NEQEHFKGER  EFNREDVVIATK  SIGVSNFSLEQLK  YTEDTTFPEGDLR  NGVTMLDTAYIYGIGR  TADVTLSQEDISFIDK  DGLVDVLQGEYNLLNR  QGNDFVFDNSPDFLKK  EANKDGLVDVLQGEYNLLNR |
| **U-2** | O31605 | Oligoendopeptidase F homolog | 77.07 | 4 | 1.78 | 109 | 85 | LYTYAHMR  LYILNHMLEGFR  QITHGNFINFLESENR  VLELDEVHIYDLYTPLVK |

Table S1B: MALDI-TOF/TOF identification of differential expressed proteins from 60 min curcumin time point using DIGE analysis

| **Spot No** | **Uniprot ID** | **Name of the protein** | **M.W (kDa)** | **No. of peptides** | **Fold change** | **Score** | **Ion Score** | **Peptides** |
| --- | --- | --- | --- | --- | --- | --- | --- | --- |
| **Down regulation** | | | | | | | | |
| D-1* | P37253 | Ketol-acid reductoisomerase | 37.43 | 19 | -1.67 | 1240 | 1163 | \| VYYNGDIK \| \| --- \| \| ESGVDVIVGVR \| \| SFTQAQEDGHK \| \| EWIVENQVNRPR \| \| VYEAEIKDELTAG \| \| VYYNGDIKENVLAGK  LIVDLMYEEGLAGMR \| \| YSISDTAQWGDFVSGPR \| \| TVAVIGYGSQGHAHALNLK \| \| FNAINASENEHQIEVVGR \| \| EAAAQAEIIMVLLPDEQQQK \| \| YSISDTAQWGDFVSGPRVVDAK \| \| TYEQGAGVPALFAIYQDVTGEAR \| \| EETETDLFGEQAVLCGGLSALK \| \| RTYEQGAGVPALFAIYQDVTGR \| \| AGFETLTEAGYQPELAYFECLHEK \| \| TVAVIGYGSQGHAHALNLKESGDVIVGVR \|   SLVFAHGFNVHFHQIVPPADVDVFLVAPK  AGVLETTFKEETETDLFGEQAVLCGGLSALVK |
| D-2* | P42973 | Aryl-phospho-beta-D-glucosidase BglA | 54.84 | 18 | -1.69 | 266 | 229 | SHIEALKK  YGMIYVDR  GYYPSYALK  YFFPDVQVR  CLRTSIGWSR  GPSVVDVMTAGAHGVPR  EGYNITFEDGDDEILR  YGMIYVDRDNEGNGSMK  EVMYQTAHHELVASALAVAK  ITDTIEENEFYPNHEAIDFYHR  KITDTIEENEFYPNHEAIDFYHR |
| D-3# | O32114 | Diaminopimelate epimerase | 30.85 | 4 | -1.72 | 109 | 100 | YAYEHK  IFNNDGSEGK  CVAKYAYEHK  VNVVTVDMGEPR |
| D-4* | P20429 | DNA-directed RNA polymerase alpha chain | 34.77 | 13 | -1.95 | 245 | 200 | IEIEKPK  SYNCLKR  FVVEPLER  LEELGLGLR  GYTPADANKR  VSYQVENTR  GYGTTLGNSLR  AKLEELGLGLR  AGINTVQELANK  FGKFVVEPLER  VLEMTIEELDLSVR  DDQPIGVIPIDSIYTPVSR  RDDQPIGVIPIDSIYTPVSR |
| D-5* | P22250 | Glutamate--tRNA ligase | 55.72 | 36 | -2.23 | 783 | 648 | VIAFNDIVK  EREEQIAR  NQGGKFIIR  SPALFDMHK  WVNNQYVK  SPALFDMHK  GEDHISNTPK  DLTQEEQEK  VYYEELLEK  TALFNYLFAR  EQFIEIFDVNR  HRDLTQEEQEK  DESIIQFIEQYK  LEELEEFTPDNIK  YAPSPTGHLHIGNAR  NIEGGEQSQLNYLK  VGTELSAEEQEWVR  CYCTEEELEKER  RDESIIQFIEQYK  GEISFESDGIGDFVIVK  AVLEEEQVPEVLSTFAAK  KLDLDQVVELTLPHLQK  DGTPTYNFAVAIDDYLMK  VAVTGQTHGPELPQSIELIGK |
| **D-6** | Q07836 | Uncharacterized protein yxxG | 16.43 | 4 | -2.72 | 274 | 207 | DFSAVLLESEYK  IKDDCIELELTPR  SNSNLFEINCNEEKLESFVTALK  GNQDSISLETIEPFLFLSIDQENGNYR |
| **D-7** | P80868 | Elongation factor G | 76.62 | 50 | -5.01 | 1120 | 889 | QATTYGVPR  GRVEGMEAR  VMTDPYVGK  ILQMHANSR  LAEEDPTFR  NIGIMAHIDAGK  CNPVLLEPIMK  VEANVGAPQVAYR  IGADFLYSVGTLR  GTRPDTNEEIER  GITITSAATTAQWK  YLEGEEITIDELK  HSSDEEPFSALAFK  QATTYGVPRIVFVNK  AMVPLAEMFGYATALR  EIPEEYKEQAEELR  EFKVEANVGAPQVAYR  EEISTVYAGDIAAAVGLK  GTFTMHMDHYEEVPK  GTLNVEFYPVLVGSAFK  KGTLNVEFYPVLVGSAFK  LFDGSYHDVDSNEMAFK  VNIIDTPGHVDFTVEVER  LTFFRVYSGTLDSGSYVK  VEVVIPEEYMGDIMGDITSR  GVQLVLDAVLDYLPAPTDVAAIK  IGETHEGASQMDWMEQEQER  DLVILESMEFPEPVIDVAIEPK  VLDGAVAVLDAQSGVEPQTETVWR  NKGVQLVLDAVLDYLPAPTDVAAIK  TQTNPETGQTIISGMGELHLDIIVDR  EYIPAVQAGLEDALENGVLAGFPLIDIK  GQFGHVWIEFEPNEEGAGFEFENAIVGGVVPR |
| **D-8** | P80868 | Elongation factor G | 76.61 | 49 | -5.92 | 1090 | 884 | QATTYGVPR  GRVEGMEAR  VMTDPYVGK  SVAEEIIKK  ILQMHANSR  LAEEDPTFR  NIGIMAHIDAGK  CNPVLLEPIMK  VEANVGAPQVAYR  IGADFLYSVGTLR  GTRPDTNEEIER  GITITSAATTAQWK  YLEGEEITIDELK  HSSDEEPFSALAFK  QATTYGVPRIVFVNK  AMVPLAEMFGYATALR  EIPEEYKEQAEELR  EFKVEANVGAPQVAYR  EEISTVYAGDIAAAVGLK  GTFTMHMDHYEEVPK  GTLNVEFYPVLVGSAFK  NIGIMAHIDAGKTTTTER  KGTLNVEFYPVLVGSAFK  LFDGSYHDVDSNEMAFK  VNIIDTPGHVDFTVEVER  LTFFRVYSGTLDSGSYVK  VEVVIPEEYMGDIMGDITSR  IGETHEGASQMDWMEQEQER  VLDGAVAVLDAQSGVEPQTETVWR  TQTNPETGQTIISGMGELHLDIIVDR  EYIPAVQAGLEDALENGVLAGFPLIDIK |
| **Up regulation** | | | | | | | | |
| **U-1** | O32201 | Protein LiaH | 25.69 | 31 | 17 | 788 | 663 | IEEMEIR  IDSESAYR  MRTLSLQK  VMLNQYVR  SAEAGTELTR  VMLNQYVR  DMESDIAKAK  EHMNTTFDK  QHTIAYQFK  KYEEAAEVAGK  QHTIAYQFKK  EFADDVEAEIEK  DMFVASVHEGLDK  IENRIEEMEIR  KEFADDVEAEIEK  ASYEQANSQLADLK  QTIVKQHTIAYQFK  IRDMFVASVHEGLDK  NQAQLAFDAGEEELAK  EHMNTTFDKIDSESAYR  ASYEQANSQLADLKEQLAALETK |
| U-2* | P21464 | 30S ribosomal protein S2 | 27.96 | 15 | 4.22 | 601 | 534 | YIFTER  RYIFTER  SGMYYVNQR  NGIYIIDLQK  KVEEAYNFTK  MQENGTFDVLPK  DLPDALFIIDPR  QLLEAGVHFGHQTR  WLGGTLTNFETIQK  DMKDLPDALFIIDPR  WLGGTLTNFETIQKR  DMKDLPDALFIIDPR  YIFTERNGIYIIDLQK  LNIPIIGIVDTNCDPDEIDVVIPANDDAIR  KLNIPIIGIVDTNCDPDEIDVVIPANDDAIR |
| **U-3** | P49814 | Malate dehydrogenase | 33.64 | 13 | 2.97 | 628 | 586 | IDAIVER  SVTQEIVK  DDLVSTNEK  ERIDAIVER  VIGQSGVLDTAR  TFVAEELNLSVK  YSYAGGIPLETLIPK  ALDMLEASPVQGFDAK  VSVIGAGFTGATTAFLIAQK  DVTGFVLGGHGDDMVPLVR  ELADVVLVDIPQLENPTK  ELADVVLVDIPQLENPTKGK  ITGTSNYEDTAGSDIVVITAGIAR |
| U-4* | P80866 | Vegetative protein 296 | 29.03 | 9 | 2.95 | 110 | 96 | MAASTLTIK  YLNEGFSGGEK  GGEFHAVMGPNGTGK  NEILQLMMIEPK  EEGDEISLMKFIR  SENFGCLMITHYQR |
| U-5* | P17820 | Chaperone protein dnaK | 66 | 29 | 2.79 | 1000 | 793 | VILVGGSTR  NEFEEIK  VIANAEGNR  TTPSVVAFK  IPAVQEAIK  AQQAQGGANAEGK  FQLTDIPPAPR  SSSGLSDEEIER  FEELSSHLVER  TTPSVVAFKNGER  AAIEKNEFEEIK  SYAESYLGETVSK  QSITNPNTIMSIK  HMGTDYKVEIEGK  AKFEELSSHLVER  AVITVPAYFNDAER  QSITNPNTIMSIKR  IINEPTAAALAYGLDK  KDELQTIVQELSMK  DYTPQEVSAIILQHLK  LGGDDFDQVIIDHLVSEFK  GVPQIEVSFDIDKNGIVNVR  VIGIDLGTTNSCVAVLEGGEPK  LGGDDFDQVIIDHLVSEFKK  GVNPDEVVALGAAIQGGVITGDVK  ADDNVVDAEYEEVNDDQNKK  QALQDAGLSASEIDKVILVGGSTR  DLSGVSSTQISLPFITAGEAGPLHLELTLTR  SQVFSTAADNQTAVDIHVLQGERPMSADNK |
| **U-6** | P54550 | Probable NADH-dependent flavin oxidoreductase yqiM | 37.58 | 14 | 2.33 | 513 | 463 | ADLIFIGR  LFTPITIK  IGIQLAHAGR  VKETVQEFK  QVWDGPLFVR  TDEYGGSPENR  LTEQVKEQGSK  GLDIADHIGFAK  ELLRDPFFAR  LTPFHMAHYISR  QLNTEIPAPVQYER  IVMSPMCMYSSHEK  AIGQVGLIIVEASAVNPQGR  ITDQDLGIWSDEHIEGFAK |
| **U-7** | P80864 | Probable thiol peroxidase | 18.31 | 10 | 2.18 | 303 | 233 | RFNEEAAK  WCGANGIDK  SVFVLDENGK  GGPVTLVGQEVK  DMSFGEAFGVYIK  WCGANGIDKVETLSDHR  VGDQAPDFTVLTNSLEEK  LGDVNVYTISADLPFAQAR  VTIISVIPSIDTGVCDAQTR  VVYAEYVSEATNHPNYEKPIEAAK |
| **U-8** | P96611 | Thioredoxin-like protein ydbP | 12.43 | 5 | 2.11 | 120 | 102 | FYADWCPDCTR  YQVMGIPSLLIFK  TPEEVTEFLSEHIS  TPEEVTEFLSEHIS  ITTNEQFNELIQSDKEIIVK |
| U-9* | P49778 | Elongation factor P | 20.47 | 7 | 2.07 | 203 | 175 | VVETEPGIK  MISVNDFR  VVDFQHVKPGK  TGLTIEVDGGIWR  VVETEPGIKGDTASGGTKPAK  TETGLVVNVPFFVNEGDTLVVNTSDGSYVSR |
| U-10* | P37809 | ATP synthase beta chain | 51.42 | 29 | 1.96 | 1350 | 1192 | ETVQGFK  IEEVVEK  LVVHRAR  EVQSTLQR  IGLFGGAGVGK  VVDLLAPYIK  TIAMASTDGVQR  YDHLPEDAFR  VSQVLGPVVDVR  FTQAGSEVSALLGR  VALTGLTMAEHFR  GSYVPVKETVQGFK  TAMVFGQMNEPPGAR  FEDGHLPEIYNAIK  ALAPEIVGEEHYAVAR  EGNDLFYEMSDSGVINK  LTEMGIYPAVDPLASTSR  DVQGQDVLFFIDNIFR  EGNDLFYEMSDSGVINK  KLTEMGIYPAVDPLASTSR  GMEAVDTGAPISVPVGDVTLGR  VFNVLGENIDLNEPVPADAK  TREGNDLFYEMSDSGVINK  QAPSFDQLSTEVEILETGIK  MPSAVGYQPTLATEMGQLQER  RIQFFLSQNFHVAEQFTGQK  ELQDIIAILGMDELGEEDKLVVHR  TVLIQELINNIAQEHGGISVFAGVGER  ISQPAASENEVGIDLTLEVALHLGDDTVR |
| **U-11** | O35022 | FMN-dependent NADH-azoreductase 1 | 22.98 | 17 | 1.78 | 436 | 391 | DMINGTFK  ENLPYLGR  LYEAFLAAYK  YTQEGPVGLMGGK  AGQGMEMTEDEKK  YTQEGPVGLMGGKK  ENNPNDEVVELDLHK  AGVTFKYTQEGPVGLMGGK  ENNPNDEVVELDLHKENLPYLGR |
| **U-12** | P28599 | 10 kDa chaperonin | 10.17 | 5 | 1.51 | 157 | 122 | MLKPLGDR  TASGIVLPDSAK  YEGTEYLILR  VVIELVESEEK  YAGTEVKYEGTEYLILR |

Table S1C: MALDI-TOF/TOF identification of differential expressed proteins from 120 min curcumin time point using DIGE analysis

| **Spot No** | **Uniprot ID** | **Name of the protein** | **M.W (kDa)** | **No. of peptides** | **Fold change** | **Score** | **Ion Score** | **Peptides** |
| --- | --- | --- | --- | --- | --- | --- | --- | --- |
| **Down regulation** | | | | | | | | |
| **D-1** | **P23630** | Diaminopimelate decarboxylase | 48.7 | 18 | -3.64 | 482 | 414 | AQVAYASK  KLDEWR  YEAAAANR  DSYSFVSK  QAFISAGLK  IHFHGNNK  ETYEDIVK  VLNLGGGFGIR  ETGHSIDVLLR  FGFDLHNGQTER  YGTPLYVYDVALIR  SLVGDAGTTLYTVGSQK  YTEDDEPLHATEYVEK  IPRPAVVFVENGEAHLVVK  QNQHGHLEIGGVDALYLAEK  ITPGVEAHTHDYITTGQEDSK  QYVAVDGGMNDNIRPALYQAK  IGCIVVDNFYEIALLEDLCK |
| **D-2** | P30950 | Delta-aminolevulinic acid dehydratase | 36.2 | 4 | -3.57 | 146 | 102 | AMREMVK  YSSEFYGPFR  AGADIIAPSNMMDGFVTVIR  EAQSDVEEGADFLIVKPSLSYMDIMR |
| **D-3** | P32395 | Uroporphyrinogen decarboxylase | 39.64 | 3 | -3.57 | 208 | 164 | ADHTPVWYMR  AIQIFDSWVGALNQADYR  YGLFEITHQPELCAYVTR |
| **D-4** | P54419 | S-adenosylmethionine synthetase | 44 | 10 | -1.67 | 104 | 83 | HGGGAFSGK  YFINPTGR  QTAAYGHFGR  HDVDLPWER  IIVDTYGGYAR  KIIVDTYGGYAR NNFDLRPAGIIK  FVIGGPQGDAGLTGR  RLFTSESVTEGHPDK  TQVTVEYDENNKPVR |
| D-5* | P39121 | Deoxyribose-phosphate aldolase | 22.19 | 6 | -2.35 | 175 | 150 | EDIALMR  DKEDDVVEADIR  TKEDVDTMVEAGASR  FASVCVNPTWVELAAK  VIIETCLLTDEEKER  SLANIIDHTALKPHTQK |
| **D-6** | P05654 | Aspartate carbamoyltransferase | 34.31 | 16 | -2.29 | 468 | 373 | SNAEVLTR  FSFEVAEK  GETLYDTIR  FSFEVAEKK  QMKNGVFIR  RGEAAYVISH  GLTVSIHGDIK  YGLTVERAER  TRFSFEVAEK  HAIIMHPAPVNR  FAANLFFEPSTR  GVEIDDSLVESEK  TLESIGVDVCVIR  MAVIQRALQTNVK  LGMNVLNLDGTSTSVQK  KLGMNVLNLDGTSTSVQK |
| **D-7** | P52996 | 3-methyl-2-oxobutanoate hydroxymethyltransferase | 30 | 3 | -1.94 | 328 | 284 | LEGGEGVFESIR  IDETIETAISGYVQDVR  ADGQVLVYHDIIGHGVER |
| D-8* | P21882 | Pyruvate dehydrogenase E1 component subunit beta | 35.47 | 26 | -1.69 | 317 | 171 | GLLISAIR  WTSPVTIR  DGISAEVVDLR  AILSLEAPVLR  VVIPSTPYDAK  SGGRWTSPVTIR  DNDPVVFLEHMK  ATEGLQKEFGEDR  QAGIAANVVAEINDR  AQMTMIQAITDALR  TVSPLDIDTIIASVEK  AADELEKDGISAEVVDLR  TVSPLDIDTIIASVEKTGR  AQMTMIQAITDALRTELK  SFRQEVPEEEYTIELGK  EGTDLSIITYGAMVHESLK  NDENVLVFGEDVGVNGGVFR  REGTDLSIITYGAMVHESLK  GLLISAIRDNDPVVFLEHMK  VAAPDTVFPFSQAESVWLPNHK  AIVVQEAQKQAGIAANVVAEINDR  TELKNDENVLVFGEDVGVNGGVFR  QAGIAANVVAEINDRAILSLEAPVLR  EGTDLSIITYGAMVHESLKAADELEK  SPFGGGVHTPELHADSLEGLVAQQPGIK  VAAPDTVFPFSQAESVWLPNHKDVLETAR |
| D-9* | Q9KWU4 | Pyruvate carboxylase | 127.9 | 30 | -2.75 | 315 | 188 | IRSHDLK  KVNIPEQK  ACTELNIR  VDVDQQPAR  GAEGLANWVK  GMTLAIDAVR  FLKEDPWK  DAHQSLLATR  IFDSLNWVK  HIEVQVIGDK  SVLLTDTTFR  QGNVVHLFER  TNIPFLENVAK  VVYFELNGQPR  EKPAFDKPLGVK  GNIGQPHGGFPEK  AAFGNDEVYVEK  DIFTIGYAIQSR  LISIGEPQPDATR  YKADEAYLVGEGK  EVPNTLFQMLLR  RCEEEGIVFIGPK  LSTWALTFEQAAAK  VIEVAPSVSLSPELR  VAEAAICYTGDILDK  GNIGQPHGGFPEKLQK  SSNAVGYTNYPDNVIK  VNDMFGDIVKVTPSSK  VVGDMALYMVQNNLTEK  GQEPITVRPGELLEPVSFEAIK |
| **D-10** | O32117 | NADH dehydrogenase-like protein yutJ | 39.75 | 19 | -2.28 | 865 | 737 | QVLFQDR  NPYHCLK  VSFPEHPR  SGLLWMYK  WFEEHGVR  GNLILSSFPER  DDLNIILFDR  DLDVEKDAQGR  LVLIGGGYGNMR  AGFGLVADRPLIGR  KAGFGLVADRPLIGR  ESRDDLNIILFDR  WNGEALPESMPQFK  RWNGEALPESMPQFK  LDVQYGDITSIDIVQK  TEYYALAAGTISDHHIR  LLPNQLPDDVSITLIDR  YHNVPGAPEFTYSIQTIDQSR  LNNLSANATVAIVGAGLSGVELASELR |
| **Up regulation** | | | | | | | | |
| **U-1** | P80244 | ATP-dependent Clp protease proteolytic subunit | 21.6 | 5 | 1.63 | 323 | 241 | AYDIYSR  TGQPLEVIER  MNLIPTVIEQTNR  MNLIPTVIEQTNRGER  YALPNSEVMIHQPLGGAQGQATEIEIAAK |
| U-2* | P21880 | Dihydrolipoyl dehydrogenase | 49.7 | 20 | 1.67 | 996 | 929 | ALINAGHR  FPFAANGR  QMSSLVTR  ALSLNETDGFMK  GEAYFVDSNSVR  TVDADYVLITVGR  ALINAGHRYENAK  VMDENSAQTYTFK  ATLGGVCLNVGCIPSK  GVEERPDGVTVTFEVK  NAIIATGSRPIELPNFK  GEEKTVDADYVLITVGR  VDVVKGEAYFVDSNSVR  HSDDMGITAENVTVDFTK  RPNTDELGLEQVGIEMTDR  VTVVEKATLGGVCLNVGCIPSK  GVEERPDGVTVTFEVKGEEK  TNVPNIYAIGDIIEGPPLAHK  VVGDFPIETDTLVIGAGPGGYVAAIR  YENAKHSDDMGITAENVTVDFTK |
| U-3* | P20429 | DNA-directed RNA polymerase alpha chain | 34.77 | 13 | 2 | 245 | 200 | IEIEKPK  SYNCLKR  FVVEPLER  LEELGLGLR  GYTPADANKR  VSYQVENTR  GYGTTLGNSLR  AKLEELGLGLR  AGINTVQELANK  FGKFVVEPLER  VLEMTIEELDLSVR  DDQPIGVIPIDSIYTPVSR  RDDQPIGVIPIDSIYTPVSR |
| **U-4** | P81101 | Ribosome recycling factor | 20.62 | 10 | 2.56 | 346 | 273 | VAVRNVR  TAIGDIEK  ANPSLLDK  LTDEYVSK  MLVITPYDK  IAIPALTEER  NGDITEDELR  LEKNGDITEDELR  ADLGLTPTSDGNMIR  VTVEYYGAQTPLNQLSSINVPEAR |
| **U-5** | O32259 | Lactate utilization protein C | 26.27 | 2 | 2.58 | 223 | 199 | TGGVAVPEWAHQPQYK  DWPSEGTPVWEWDADKGEENIK |
| U-6* | P37487 | Manganese-dependent inorganic pyrophosphatase | 34 | 7 | 2.6 | 379 | 283 | LGFNAEPVR  IANFETAEPLYYR  DIEEVQVLEVIDHHR  ELAEIAGVDAEEYGLNMLK  LVETAANEVNGVILVDHNER  QQSIKDIEEVQVLEVIDHHR  ILIFGHQNPDTDTICSAIAYADLK |
| **U-7** | P28598 | 60 kDa chaperonin | 57.25 | 25 | 2.84 | 1340 | 1224 | ALEEPIR  APGFGDRR  GTFNAVAVK  GVDALADAVK  STQIAQLGR  IEDALNSTR  NVTAGANPVGVR  LRIEDALNSTR  AQVEETTSEFDR  GYASPYMVTDSDK  FGSPLITNDGVTIAK  EIELEDAFENMGAK  KGMEQAVAVAIENLK  AQVEETTSEFDREK  QIAHNAGLEGSVIVER  MEAVLDNPYILITDK  GFTTELEVVEGMQFDR  ENTTIVEGAGETDKISAR  VAAVEAEGDAQTGINIVLR  AAVEEGIVSGGGTALVNVYNK  TNDVAGDGTTTATVLAQAMIR  AMLEDIAVLTGGEVITEDLGLDLK  LKNEEIGVGFNAATGEWVNMIEK  TNDVAGDGTTTATVLAQAMIREGLK  ESIAQVAAISAADEEVGSLIAEAMER |
| **U-8** | P15874 | GrpE protein (HSP-70 cofactor) | 21.53 | 6 | 3.98 | 186 | 167 | VQADFENYK  SRLEMEASQK  VQADFENYKR  SLLQGMEMVHR  ALQVEADNEQTK  SQNIVTDLLPALDSFER |
| U-9* | P37869 | Enolase | 46.42 | 8 | 5.86 | 75 | 49 | YNQLLR  AGYTAVISHR  PYIVDVYAR  MGAQIFHSLK  LGANAILGVSMACAR  VQLVGDDLFVTNTK  ALVPSGASTGEYEAVELR  GNPTVEVEVYTETGAFGR |
| U-10* | P39121 | Deoxyribose-phosphate aldolase | 23.47 | 13 | 1.72 | 611 | 524 | EDIALMR  TVGPDIGVK  IGASAGVSIVK  LAVSAGADFVK  TSTGFSTGGATK  DKEDDVVEADIR  GATEVDMVINIAALK  TKEDVDTMVEAGASR  GATEVDMVINIAALKDK  VIIETCLLTDEEKER  SLANIIDHTALKPHTQK  TSTGFSTGGATKEDIALMR  GTGVDVCTVIGFPLGANTTETK |
| **U-11** | P09339 | Aconitate hydratase | 99.61 | 28 | 4.13 | 789 | 664 | QVDGFVIK  VLLESVLR  DFNSYGSR  GNHEVMMR  TVIAESFER  QTVTPELFR  HGGILQMVLR  RGNHEVMMR  DIDVPFKPSR  LPNGTTATDLALK  NDLLITSVLSGNR  EYETVFDDNKR  FDSEVEIDYYR  SNLVFMGVLPLQFK  DPEHIDVVEAYCR  WNEIETTDEALYK  EVIEVDVDETVRPR  AINEDGNVTTFEAVVR  GNHEVMMRGTFANIR  FGDSVTTDHISPAGAIGK  RPQDLIPLSAMQETFK  FVEFFGPGIAELPLADR  AGTEDALAVNMDLEFER  RPQDLIPLSAMQETFKK  LPNGTTATDLALKVTQVLR  VILQDFTGVPAVVDLASLR  RPQDLIPLSAMQETFKK  LTGRDPEHIDVVEAYCR |

- # : Proteins unique in DIGE
- Bold : Same trend in both DIGE and iTRAQ
- * No significant change in iTRAQ (less than 1.2 fold up and down)
