# Supplementary material for: Comprehensive Analysis of Temporal Alterations in Cellular Proteome of Bacillus subtilis under Curcumin Treatment
Source: PLoS One. 2015 Apr 14;10(4):e0120620. doi: 10.1371/journal.pone.0120620 (PMC4397091; doi:10.1371/journal.pone.0120620)
Supplement: S4 Table — (DOCX) [file pone.0120620.s011.docx]

Table S4: DIGE labelling strategy for curcumin time point analysis

| **Time point** | **Cy3** | **Cy5** | **Internal control (Cy2)** |
| --- | --- | --- | --- |
| 20 min | Control-1 | 20 min-1 | All samples |
|  | Control-2 | 20 min-2 | All samples |
|  | 20 min-3 | Control-3 | All samples |
| 60 min | Control-1 | 60 min-1 | All samples |
|  | Control-2 | 60 min-2 | All samples |
|  | 60 min-3 | Control-3 | All samples |
| 120 min | Control-1 | 120 min-1 | All samples |
|  | Control-2 | 120 min-2 | All samples |
|  | 120 min-3 | Control-3 | All samples |
