# Supplementary figures and images for: Correction: Comprehensive Analysis of Temporal Alterations in Cellular Proteome of Bacillus subtilis under Curcumin Treatment
Source: PLoS One. 2015 Jun 17;10(6):e0130782. doi: 10.1371/journal.pone.0130782 (PMC4471116; doi:10.1371/journal.pone.0130782)

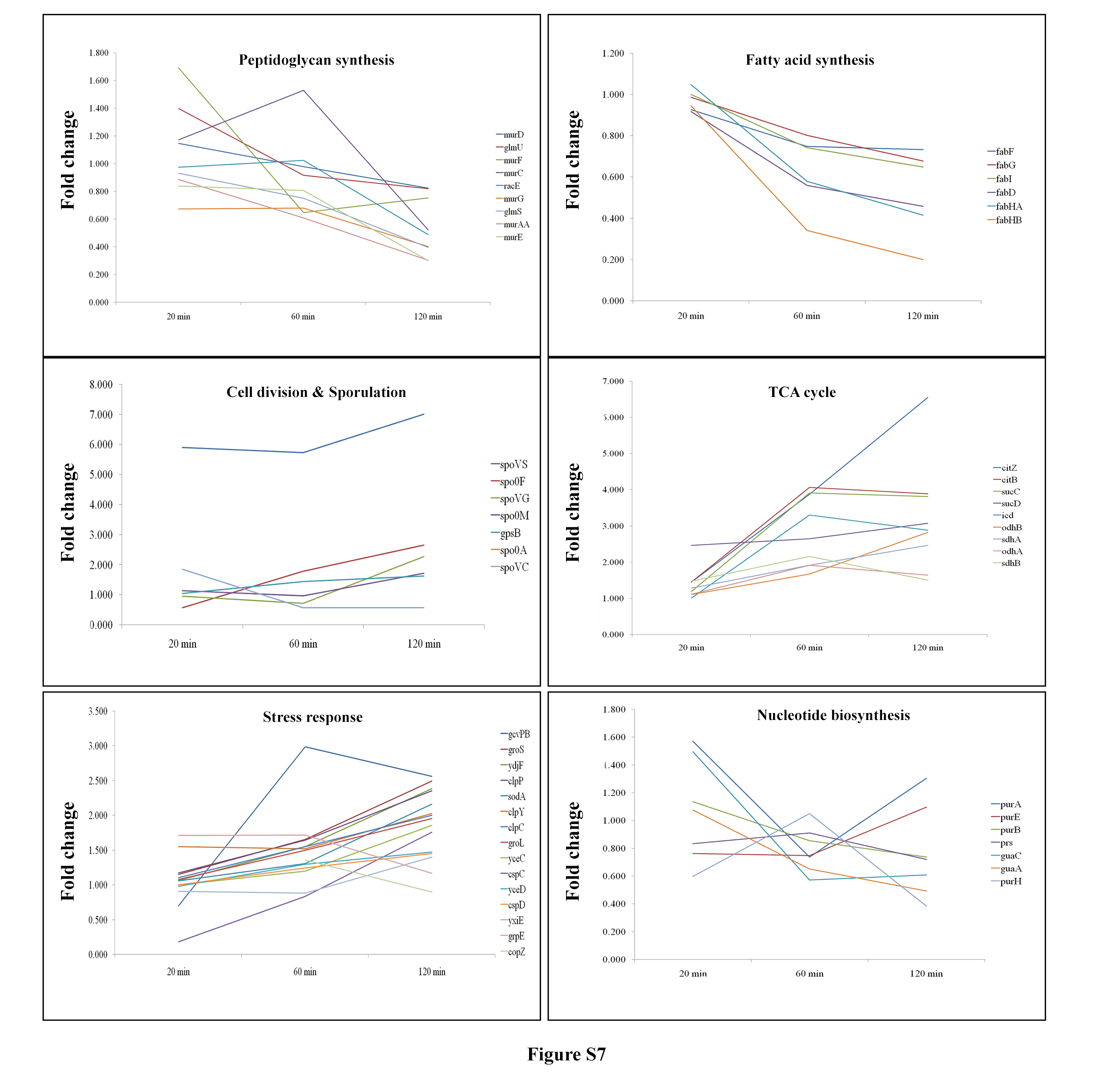

Supplement: S7 Fig — (TIF) [file pone.0130782.s001.tif]
